# Thermally Activated Shrinkage of Heat-Shrinkable Fibers and Its Influence on Drying Shrinkage and Mechanical Properties of Cement Mortar

**DOI:** 10.3390/polym18212776

**Published:** 2026-11-06

**Authors:** Qihang Zhou, Haobing Fu, Ningyang Feng, Tusheng He, Huaqiang Cheng, Jie Chen, Jinhui Li, Zi Yu, Wei Du, Leheng Li, Qingjun Ding

**Affiliations:** 1 College of Materials Science and Engineering, Wuhan Textile University, Wuhan 430200, China; 723571605zqh@gmail.com (Q.Z.); yu1018541283@163.com (Z.Y.); duwei@wtu.edu.cn (W.D.); 2 State Key Laboratory of New Textile Materials and Advanced Processing Technologies, Wuhan 430200, China; 3 Poly Changda Engineering Co., Ltd., Guangzhou 510510, China; fuhaobing1105@163.com; 4 Xinjiang Xiyu Highway Construction Group Co., Ltd., Urumqi 830000, China; 18099177585@163.com (N.F.); 13094042297@163.com (J.C.); 5 School of Materials Science and Engineering, Wuhan University of Technology, Wuhan 430070, China; hts0539@163.com (T.H.); 18186221227@163.com (L.L.); 6 Wuhan Municipal Construction Group Co., Ltd., Wuhan 430200, China; huaqiangcheng@163.com

**Keywords:** heat-shrinkable fibers, thermal shrinkage, cement mortar, mechanical properties, drying shrinkage, crack-mitigation potential

## Abstract

High-strength concrete applied in mass concrete structures typically features low water-to-binder ratios and high binder contents, resulting in intense hydration heat evolution and pronounced shrinkage deformation; when coupled with its inherently limited tensile strain capacity, the material is highly susceptible to cracking, thereby compromising long-term structural durability. To actively mitigate shrinkage deformation and reduce cracking risk, high-modulus heat-shrinkable fibers were incorporated into cementitious materials. Their thermal shrinkage behavior and the resulting effects on the mechanical properties and shrinkage performance of mortar were systematically investigated. The results showed that the fibers exhibited a pronounced thermally activated shrinkage response within the temperature range of 50–90 °C, with tensile strength exceeding 600 MPa and elastic modulus greater than 9 GPa. The shrinkage ratio increased significantly with temperature and reached 2.80% at 90 °C, corresponding to a maximum calculated recovery stress of 14.79 MPa. Temperature was identified as the dominant factor governing fiber shrinkage behavior, whereas the influence of initial fiber length was negligible. Incorporation of heat-shrinkable fibers effectively enhanced the overall performance of mortar. An optimum fiber length of 12 mm and volume fraction of 0.3% provided the best balance between strength development and shrinkage reduction. Under these conditions, the 28-day flexural and compressive strengths exceeded 12.5 MPa and 75.0 MPa, respectively, while the 28-day drying shrinkage was reduced by 49.5% in the dosage-screening series and by 54.7% in the independent validation series (233 με); the lowest absolute 28-day shrinkage (220 με) was obtained at 0.5% fiber content, but at the cost of flowability and compressive strength. Under steam curing the fibers were activated further and the mortar reached 13.2 MPa and 77.8 MPa; the same regime did not raise the strength of the plain matrix above its standard-cured value. The significant reduction in drying shrinkage and improved flexural behavior is attributed to the combined effects of fiber bridging and thermally induced shrinkage stress. The contraction of activated fibers is inferred to induce localized pre-compressive effects within the matrix, which can help counteract shrinkage-induced tensile stress, thereby reducing the driving force for early-age microcracking.

## 1. Introduction

Modern concrete infrastructure, including long-span bridges, offshore platforms, immersed tunnels, nuclear power plants, and large-scale industrial foundations, increasingly relies on high-strength cementitious materials because of their superior mechanical performance, excellent durability, and reduced permeability [1,2,3]. The widespread adoption of low water-to-binder ratios and high cementitious material contents has significantly improved the structural capacity and service life of concrete structures [1,2], enabling the construction of increasingly larger and more complex engineering systems [4]. Meanwhile, to shorten construction cycles and satisfy the demand for rapid strength development, accelerated curing techniques, particularly steam curing, have been extensively employed in precast concrete production and massive concrete construction [4,5]. However, these advances have also introduced new challenges associated with early-age performance. The rapid heat evolution induced by cement hydration, together with elevated curing temperatures and restrained volumetric deformation, considerably increases the susceptibility of high-strength cementitious materials to early-age cracking. Such cracks often develop before the material reaches its design strength and subsequently provide preferential pathways for the ingress of water and aggressive substances [6,7,8], thereby compromising long-term durability and structural reliability [9,10]. Consequently, effective mitigation of early-age cracking has become one of the most important research priorities in the development of durable cementitious materials, particularly for high-strength concrete subjected to elevated curing temperatures. Understanding the underlying mechanisms of crack initiation and propagation is therefore essential for developing next-generation crack-control technologies capable of improving the durability and service performance of modern concrete infrastructure [1,6,7].

Early-age cracking in high-strength cementitious materials originates from the coupled effects of hydration heat evolution, volumetric deformation, and restraint conditions, which become increasingly pronounced as concrete strength and structural dimensions continue to increase. Compared with conventional concrete, high-strength cementitious materials generally possess lower water-to-binder ratios and higher binder contents to achieve superior mechanical properties and durability. However, these characteristics also accelerate cement hydration and intensify self-desiccation, resulting in significant autogenous shrinkage at early ages [11,12]. Simultaneously, the exothermic hydration of cement generates considerable internal heat, leading to rapid temperature rise within the material [13]. As hydration gradually slows, subsequent cooling induces thermal contraction, while the concurrent development of autogenous and drying shrinkage further increases tensile deformation [14,15]. Under practical engineering conditions, these volumetric changes are inevitably restrained by aggregates, reinforcement, adjacent structural components, or differential temperature distributions [16], causing tensile stresses to accumulate continuously before the concrete has developed sufficient tensile strength. Once the tensile stress exceeds the early-age tensile capacity of the cementitious matrix, microcracks initiate and gradually propagate into visible cracks, thereby compromising the integrity and long-term durability of the structure [14,16].

The cracking risk is particularly critical for mass concrete and steam-cured cementitious materials, where elevated curing temperatures significantly alter hydration kinetics and thermal stress development. Steam curing is widely employed in precast concrete production and high-performance concrete construction because it accelerates hydration reactions and enables rapid strength development, thereby improving construction efficiency and shortening production cycles. Nevertheless, elevated curing temperatures also intensify temperature gradients, accelerate moisture redistribution, and amplify the mismatch between thermal contraction and restrained deformation during the cooling stage. Consequently, concrete subjected to steam curing often experiences higher thermal stresses and more severe shrinkage-induced cracking than concrete cured under ambient conditions. These phenomena become increasingly significant in high-strength systems because their dense microstructure limits internal stress relaxation and increases sensitivity to thermal and shrinkage deformation. Therefore, mitigating the coupled effects of temperature rise, thermal contraction, and early-age shrinkage under elevated curing conditions remains a fundamental challenge for improving the durability of high-strength cementitious materials [17,18].

To mitigate early-age thermal and shrinkage-induced cracking, extensive research has been devoted to the development of crack-control technologies from both structural and material perspectives. Existing approaches can generally be classified into four categories: temperature regulation, shrinkage-compensating systems, internal curing technologies, and fiber reinforcement. Temperature regulation methods, including optimized casting procedures, insulation systems [19,20], cooling pipes [21], and staged construction [22,23], are widely adopted in mass concrete engineering to reduce temperature gradients and restraint stresses [24]. Although these measures effectively moderate thermal development, they are highly dependent on construction management, require additional equipment and operational costs, and mainly control the external thermal environment rather than fundamentally improving the intrinsic crack resistance of cementitious materials [22,25].

Material-based crack-control strategies have therefore attracted increasing attention because they directly modify the deformation characteristics of cementitious composites. Among them, mineral expansive agents, particularly magnesium oxide (MgO)- and calcium sulfoaluminate (CSA)-based expansive systems, have been extensively investigated for compensating autogenous and drying shrinkage through the formation of expansive hydration products. Nevertheless, their expansion behavior is strongly influenced by curing temperature, hydration conditions, restraint level, and dosage. Excessive expansion may introduce internal microcracking, whereas insufficient expansion fails to provide adequate shrinkage compensation, making accurate expansion control difficult under practical engineering conditions.

Internal curing has emerged as another effective approach for mitigating self-desiccation and autogenous shrinkage by supplying additional curing water during cement hydration. Saturated lightweight aggregates [26,27,28] and superabsorbent polymers (SAPs) [29,30,31] are the most commonly adopted internal curing materials because they gradually release stored water as hydration proceeds, thereby maintaining internal relative humidity and reducing capillary stress [31,32]. Although internal curing significantly decreases autogenous shrinkage and promotes continued hydration, the formation of additional macropores after water release may adversely affect mechanical properties when the dosage or particle characteristics are not properly optimized [29,30]. Furthermore, the effectiveness of internal curing gradually diminishes under severe restraint conditions where tensile stresses are primarily governed by thermal contraction rather than moisture loss [26,32].

Overall, despite the considerable progress achieved by these technologies, their crack-control mechanisms remain essentially indirect. Existing approaches primarily reduce temperature rise, compensate shrinkage, or alleviate internal moisture loss, but they generally do not actively generate compressive stress to offset tensile stress development during the critical early-age period [22,24]. Consequently, the effectiveness of these methods is often limited in high-strength cementitious materials subjected to elevated curing temperatures, where rapid hydration, restrained deformation, and thermal contraction occur simultaneously [33,34,35]. These limitations have stimulated growing interest in developing active reinforcement strategies capable of directly compensating tensile stresses during the early stages of concrete hardening.

Fiber reinforcement has been widely recognized as one of the most effective material-based approaches for improving the crack resistance and durability of cementitious composites. Depending on the material type and mechanical properties, steel fibers [36,37,38], polypropylene (PP) fibers [39,40,41], polyvinyl alcohol (PVA) fibers [42,43], basalt fibers, glass fibers [44], carbon fibers, and medium-high molecular weight polyethylene (MHMWPE) fibers [36,45] have all been incorporated into cement-based materials to mitigate crack development and enhance post-cracking performance. These fibers improve the mechanical behavior of concrete primarily by bridging microcracks, transferring tensile stresses across crack surfaces, delaying crack propagation, and increasing the energy absorption capacity of the composite. As a result, significant improvements in tensile strength, flexural toughness, impact resistance, fatigue performance, and long-term durability have been widely reported [46,47].

Among various synthetic fibers, polypropylene fibers have been extensively adopted in engineering practice owing to their good chemical stability, low density, low cost, and resistance to corrosion [41,48]. Numerous studies have demonstrated that PP fibers effectively reduce plastic shrinkage cracking and improve crack distribution by forming a three-dimensional reinforcing network within the cement matrix [49,50]. Similarly, PVA fibers exhibit strong interfacial bonding with cement hydration products and are capable of promoting multiple microcracking behavior, thereby significantly improving the ductility and strain-hardening characteristics of engineered cementitious composites (ECC) [42,43,46]. MHMWPE fibers, characterized by their exceptionally high tensile strength, high elastic modulus, and superior chemical resistance, have also attracted increasing attention for developing high-performance cementitious composites with enhanced crack resistance and durability [51,52].

Despite their demonstrated effectiveness, the reinforcing mechanisms of conventional fibers remain fundamentally passive [53,54]. The beneficial effects of these fibers are primarily mobilized only after tensile deformation or microcrack initiation has already occurred within the surrounding cement matrix [51,53]. Their contribution relies mainly on crack bridging, fiber pull-out resistance, stress redistribution, and energy dissipation during crack propagation, rather than preventing the initial development of tensile stresses [24,33]. Consequently, although conventional fibers can effectively restrict crack width and delay crack propagation, they are generally unable to actively compensate for the tensile stresses generated by hydration heat, autogenous shrinkage, drying shrinkage, or thermal contraction during the early-age stage of cementitious materials. This intrinsic limitation becomes particularly pronounced in high-strength mass concrete subjected to elevated curing temperatures, where large thermal gradients and restrained shrinkage produce significant tensile stresses before conventional fibers can fully exert their reinforcing effect.

Therefore, developing a new generation of fiber reinforcement capable of actively generating internal compressive stress during the early-age period has become an emerging research direction. In recent years, shape-memory polymers (SMPs) [55,56,57,58,59,60] capable of undergoing reversible entropic recovery in response to external thermal stimuli have attracted significant interest across materials science and smart infrastructure [55,57,58]. Through tailored molecular architecture and crosslinked network design, these stimuli-responsive polymers can store temporary deformation and subsequently release stored mechanical strain upon heating [59,60].

The concept of utilizing shrinkable polymers to actively manage stresses in cementitious systems was originally demonstrated by Jefferson et al. [61], Isaacs et al. [62], and Teall [63], who embedded prestressed shape-memory polymer tendons into concrete beams to achieve post-cracking crack closure and enhance autogenous healing upon external thermal activation. Similar shape-memory mechanisms have also been applied to joint sealants [64] and self-healing matrices [65]. Furthermore, polymer blend compatibilization and phase morphology control have proven vital in tuning the recovery force and transition kinetics of such responsive systems [56,66,67].

However, existing smart-tendon approaches have primarily functioned as macro-scale remedial systems designed to close already-developed cracks via external heating. In contrast, incorporating discrete, micro-dispersed heat-shrinkable fibers (HSFs) directly into the cementitious matrix provides a fundamentally proactive crack-mitigation strategy. By leveraging the intrinsic hydration heat or standard steam curing of the material, uniformly distributed HSFs contract during early-age hardening, thereby generating internal pre-compressive restraint to offset tensile stress buildup before microcracks coalesce.

Beyond the structural scale of reinforcement, a fundamental materials-science limitation of existing shape-memory polymer (SMP) fiber systems lies in their intrinsic trade-off between actuation capability and mechanical stiffness. Conventional SMP fibers documented in the literature—such as polyurethane-, PLA-, or uncrosslinked polyester-based systems—typically possess modest elastic moduli (<2.5 GPa) and low tensile strengths (<250 MPa) [55,58,66]. When embedded in high-strength cementitious composites characterized by a rigid matrix modulus (30–40 GPa), such low-modulus fibers undergo excessive elastic elongation under stress and exhibit poor crack-bridging resistance, substantially compromising their load-transfer efficiency.

To fundamentally overcome this mechanical bottleneck, the current work develops a novel polymer composite architecture based on a metallocene polypropylene/medium-high molecular weight polyethylene (mPP/MHMWPE) binary blend synergized with dual-agent radiation crosslinking. In this system, the highly stereoregular mPP phase serves as a rigid crystalline skeleton sustaining high axial loads, while the dispersed MHMWPE phase acts as the thermally responsive entropic contraction network. A single crosslinking agent cannot balance the distinct radical reaction kinetics of PP (which predominantly degrades via chain scission) and PE (which crosslinks readily). By introducing a synergistic dual-crosslinking formulation—trimethylolpropane trimethacrylate (TMPTMA, selectively sensitizing PP crosslinking) and triallyl isocyanurate (TAIC, sensitizing PE)—combined with electron-beam irradiation (100 kGy) and high-ratio hot drawing (6.5:1), chain slippage is suppressed while preserving molecular orientation. This design decouples high thermal shrinkage capability from load-bearing stiffness: the tensile strength exceeds 600 MPa, the elastic modulus exceeds 9 GPa, and the isometric recovery stress exceeds 14 MPa.

Although a recent exploratory study by the authors investigated the macro-scale engineering synergy between HSFs and internal curing porous aggregates in structural concrete [68], the fundamental materials science governing the HSF itself has not yet been elucidated. Specifically, the dual-monomer radiation crosslinking mechanism, molecular orientation kinetics, single-fiber recovery stress evolution, and the baseline mechanical reinforcing thresholds in cement mortar remain unaddressed. To establish this essential scientific foundation, the present study systematically examines the chemical functionalization, microstructural crystallization, and thermomechanical activation kinetics of newly engineered PP/MHMWPE heat-shrinkable fibers, providing a validated material platform for active shrinkage control in cementitious composites.

## 2. Materials and Methods

### 2.1. Materials and Design Mechanism of HSFs

#### 2.1.1. Cementitious Materials

**Figure 1:**
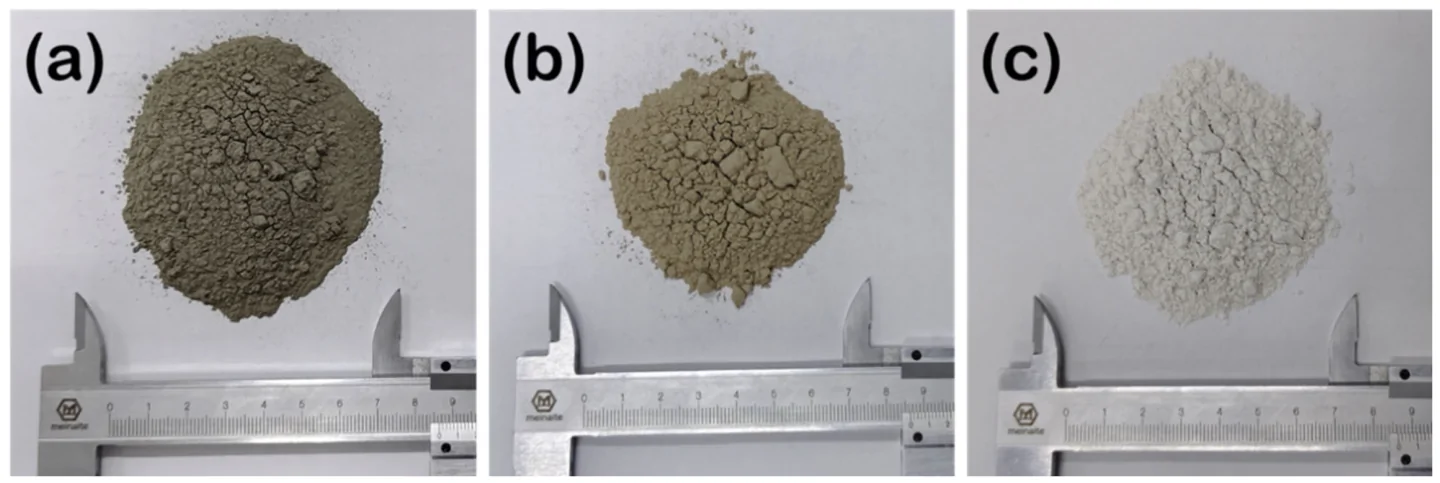
Appearance and topography of cementitious materials: (**a**) Ordinary Portland cement (P·II 52.5); (**b**) Class I fly ash (FA); (**c**) S95 ground granulated blast furnace slag (GGBFS).

Ordinary Portland cement (P·II 52.5), conforming to Chinese standard GB 175-2020 [69], was used as the primary binder. Its characteristic grey appearance is shown in Figure 1a. Class I fly ash (FA) and S95 ground granulated blast furnace slag (GGBFS) served as supplementary cementitious materials (SCMs). Their physical appearances are presented in Figure 1b and Figure 1c, respectively.

The chemical compositions and physical properties of these binders are consolidated in Table 1 and Table 2. The cement had an apparent density of 3.16 g/cm^3^ and a Blaine specific surface area of 340 m^2^/kg. Its initial and final setting times were 145 min and 210 min, respectively, with a 28-day compressive strength of 61.2 MPa.

The fly ash exhibited a specific surface area of 442 m^2^/kg, while the GGBFS measured 463 m^2^/kg. For the chemical analysis, X-ray fluorescence (XRF) was used to determine the chemical compositions. As detailed in Table 1, the fly ash is highly siliceous, typical of Class I FA, contributing to long-term pozzolanic reactions and microstructural densification [70,71,72,73]. Meanwhile, the GGBFS possesses a high calcium-to-silica ratio, indicative of stable latent hydraulic activity, which facilitates the continuous formation of calcium silicate hydrate (C-S-H) gels and refinement of the pore structure [74,75,76,77,78]. The incorporation of these supplementary cementitious materials effectively reduces early hydration heat while maintaining long-term mechanical strength [71,79,80].

#### 2.1.2. Aggregates, Water, and Admixture

Fine aggregates consisted of natural medium sand with a fineness modulus of 2.65, an apparent density of 2620 kg/m^3^, and a water absorption of 1.2%.To achieve the desired workability of the mixtures, a polycarboxylate ether (PCE)-based superplasticizer (Sobute New Materials Co., Ltd., Nanjing, China) was adopted. It featured a 40% solid content and a 30% water-reducing ratio. Potable tap water was used for specimen preparation and curing.

#### 2.1.3. Preparation and Shape-Memory Mechanism of HSFs

Heat-shrinkable fibers (HSFs) were fabricated through a melt-spinning and multi-stage drawing process using a highly-engineered polymer blend, wherein high-polymer metallocene polypropylene (mPP) serves as the load-bearing crystalline matrix to sustain mechanical deformation, and medium-high molecular weight polyethylene (MHMWPE) acts as the thermally-responsive shrinkage network.

The basic physical, macromolecular, and thermal characteristics of the virgin polymer resins were as follows:

High-polymer metallocene polypropylene (mPP): The mPP resin possessed an apparent density of 0.905 g/cm^3^ (ISO 1183 [81]) and a melt flow rate (MFR) of 14.5 g/10 min (tested at 230 °C under a 2.16 kg load per ISO 1133 [82]). High-temperature gel permeation chromatography (HT-GPC, in 1,2,4-trichlorobenzene at 150 °C) revealed a weight-average molecular weight (Mw) of approximately 2.8 × 10^5^ g/mol with a narrow polydispersity index (Mw/Mn = 2.3) and high isotacticity (>98%), providing an initial DSC melting peak temperature of Tm = 158.2 °C and a baseline crystallinity of 48.5%. This stereoregularity favours a well-oriented crystalline texture and a high axial stiffness upon drawing.

Medium-high molecular weight polyethylene (MHMWPE): The MHMWPE component exhibited an apparent density of 0.942 g/cm^3^ and a significantly lower MFR of 0.45 g/10 min (190 °C, 2.16 kg), reflecting an elevated molecular weight of Mw ≈ 6.2 × 105 g/mol (Mw/Mn = 4.6). It displayed a melting peak at Tm = 133.5 °C and an initial crystallinity of 56.2%. The extended macromolecular chains and high entanglement density of MHMWPE form an ideal elastic shrinkage network capable of storing substantial entropic orientation energy during hot drawing.

The HSFs were prepared from a blend of mPP and MHMWPE (80:20 by weight) compatibilized with 4.0 wt.% PP-g-MAH (grafting ratio ~ 1.0%) [83]. Trimethylolpropane trimethacrylate (TMPTMA, 1.0 wt.%) and triallyl isocyanurate (TAIC, 0.5 wt.%) (Sinopharm Chemical Reagent Co., Ltd., Shanghai, China) were added as radiation-crosslinking agents. The mixture was compounded using a twin-screw extruder at 180–210 °C and pelletized. The pellets were melt-spun into precursor filaments at 210 °C and quenched in a 20 °C water bath. The filaments were then irradiated by an electron beam under nitrogen at an absorbed dose of 100 kGy (acceleration voltage: 1.5 MeV). Following irradiation, the fibers were hot-drawn in an oven at 90 °C to a draw ratio of 6.5:1, passed through a pinned roller for fibrillation, and quenched in water at 15 °C to fix the oriented structure. The resulting monofilaments had an average diameter of 0.20 ± 0.01 mm (ranging from 0.18 to 0.22 mm). For the drawing-temperature study, precursor filaments were drawn at 50–90 °C; unless otherwise stated, all other tests used the 90 °C-drawn HSFs.

For surface modification, the fibers were first fed through patterned rollers at 80 °C (line pressure: 0.8 MPa) to imprint surface indentations (depth: 15–20 μm, pitch: 0.5 mm) for mechanical interlocking. The embossed fibers were then immersed in a 1.0 wt.% γ-aminopropyltriethoxysilane (γ-APS, KH-550, Sinopharm Chemical Reagent Co., Ltd., Shanghai, China) ethanol/water solution (90:10 by volume, pre-hydrolyzed at pH 4.5) for 15 min, followed by drying at 80 °C for 1 h [84,85]. Finally, the fibers were coated with a 0.3 wt.% non-ionic surfactant (polyoxyethylene fatty alcohol ether, AEO-9) aqueous solution and dried at 60 °C to facilitate dispersion in the cement mixture [86].

The shape-memory behavior of the HSFs originates from the highly oriented molecular chains generated during the drawing process. During fiber fabrication, large tensile deformation stores internal orientation energy within the polymer chains. When exposed to elevated temperatures, the molecular chains gradually relax toward their thermodynamically stable configuration, releasing the stored orientation stress and producing axial shrinkage of the fibers. This thermally induced shrinkage generates recovery stress, which can introduce compressive restraint into the surrounding cement matrix and thereby compensate for tensile stresses induced by thermal contraction. Consequently, HSFs effectively mitigate crack initiation and propagation in concrete.

**Figure 2:**
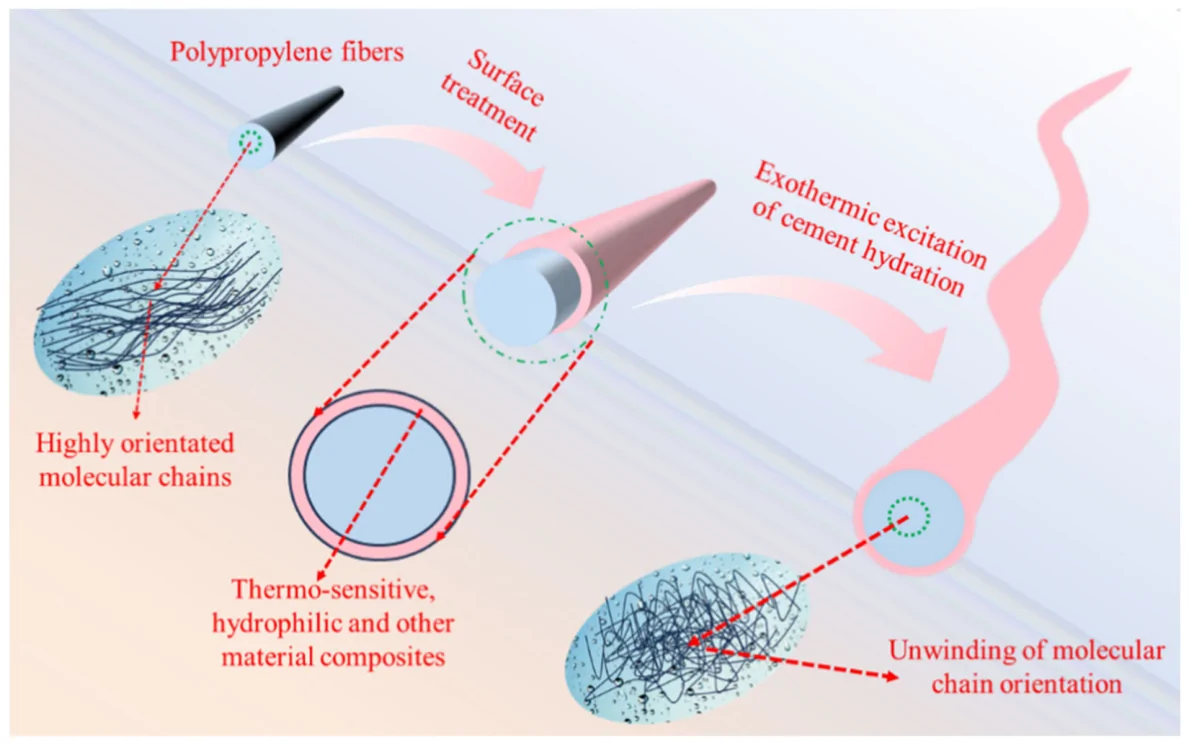
Schematic diagram of thermally activated shrinkage of heat-shrinkable fibers (dashed circles represent schematic insets showing the highly oriented molecular chain structure before activation and the entropic recoiling/relaxation of molecular chains upon hydration-heat excitation, respectively).

To characterize their thermal response, the thermal stretching and shrinkage behaviors of the HSFs were systematically investigated. Particular attention was paid to the evolution of the calculated recovery stress with temperature, providing a basis for evaluating their crack-control potential in concrete. The corresponding shape-memory mechanism is schematically illustrated in Figure 2.

**Figure 3:**
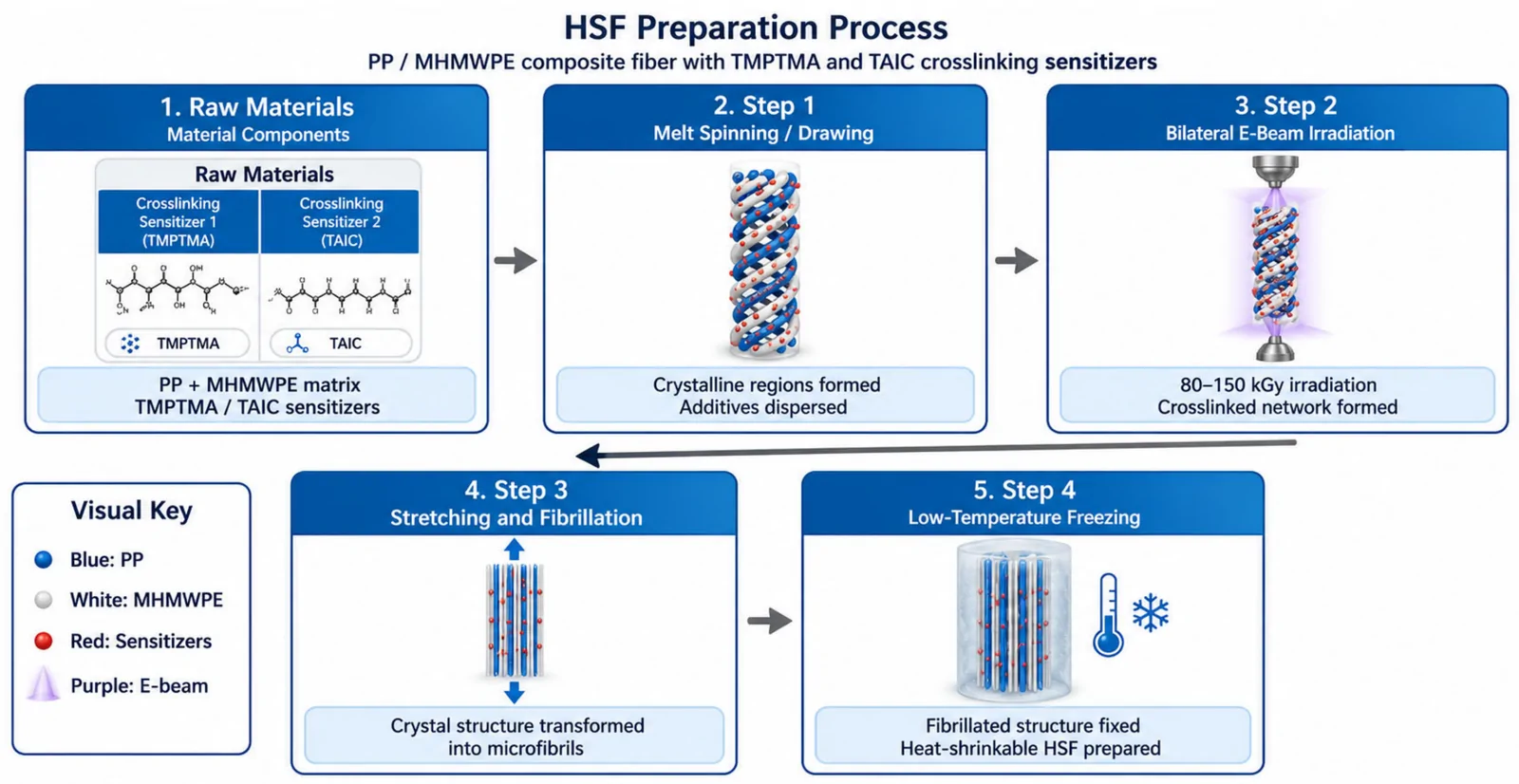
Schematic illustration of the fabrication process, radiation-induced crosslinking, molecular orientation, and thermally activated shape-memory mechanism of HSFs.

To elucidate the temperature-dependent shrinkage behavior of the heat-shrinkable fibers (HSFs), their internal shape-memory mechanism was investigated, as schematically illustrated in Figure 3. The thermally activated shrinkage originates from a radiation-induced crosslinked network together with a highly oriented polymer structure established during the fabrication process [87].

The HSFs were prepared from a polymer blend consisting of polypropylene (PP) and medium-high molecular weight polyethylene (MHMWPE), with trimethylolpropane trimethacrylate (TMPTMA) and triallyl isocyanurate (TAIC) introduced as radiation crosslinking agents. During melt spinning and drawing, the polymer chains became highly oriented along the fiber axis while maintaining their crystalline morphology. Subsequently, electron-beam irradiation induced extensive covalent crosslinking throughout the polymer matrix, forming a stable three-dimensional network that served as the permanent phase of the shape-memory structure.

Owing to the intrinsic differences in the molecular chain structures of polypropylene (PP) and polyethylene (PE), employing a single crosslinking agent cannot readily achieve an optimal and balanced crosslinking density in both phases simultaneously. In this formulation, trimethylolpropane trimethacrylate (TMPTMA) preferentially enhances the crosslinking efficiency of the PP phase. Meanwhile, triallyl isocyanurate (TAIC), due to its unique allylic molecular structure, exhibits superior crosslinking sensitization toward the PE phase. The combination of TMPTMA and TAIC is intended to give a uniform covalent crosslinked network in both phases. This dual-sensitized network effectively stabilizes the ‘shape-memory permanent phase’ of the fibers, which is critical for preserving their structural integrity and generating stable recovery stress during thermal activation.

Following crosslinking, the fibers underwent further stretching and fibrillation to introduce a high degree of molecular orientation. Rapid cooling was then applied to fix the highly oriented configuration as a temporary metastable state. Consequently, a considerable amount of orientation strain was stored within the polymer chains.

Upon heating within the thermal activation range of 50–90 °C (with 50 °C representing the onset of activation, T_onset_), molecular mobility increased, allowing the oriented polymer chains to relax toward their thermodynamically stable configuration. Owing to the constraint imposed by the radiation-induced crosslinked network, complete molecular relaxation was prevented, resulting instead in axial shrinkage of the fibers and the generation of thermal recovery stress. When embedded in concrete, this recovery stress acts as an internal prestressing force that compensates for tensile stresses caused by thermal contraction and early-age deformation, thereby effectively suppressing crack initiation and propagation.

### 2.2. Test Methods

(1) Tensile properties and elastic modulus testing of fibers

The tensile properties of the HSFs were evaluated in accordance with ASTM D3822/D3822M [88] (Standard Test Method for Tensile Properties of Single Textile Fibers) using an Instron universal testing machine (Model 5967, Instron, Norwood, MA, USA) equipped with a 50 N high-precision load cell and pneumatic yarn grips. Prior to testing, all fiber specimens were conditioned at 23 ± 2 °C and 50 ± 5% relative humidity for at least 24 h. The gauge length was set to 50 mm, and the crosshead displacement rate was maintained at 10 mm/min. Five valid replicate specimens were tested for each condition (n = 5). The elastic modulus was determined from the slope of the initial linear-elastic region of the tensile stress-strain curve within the axial strain range of 0.05–0.25%. To evaluate stiffness retention after thermal activation, fibers were placed in a constant-temperature water bath at designated temperatures (40, 50, 60, 70, 80, and 90 °C) for 30 min in an unrestrained state, cooled to ambient temperature (23 °C) for 2 h, and then tested at room temperature under the same tensile protocol.

(2) Free Thermal Shrinkage Ratio Test of Fibers

**Figure 4:**
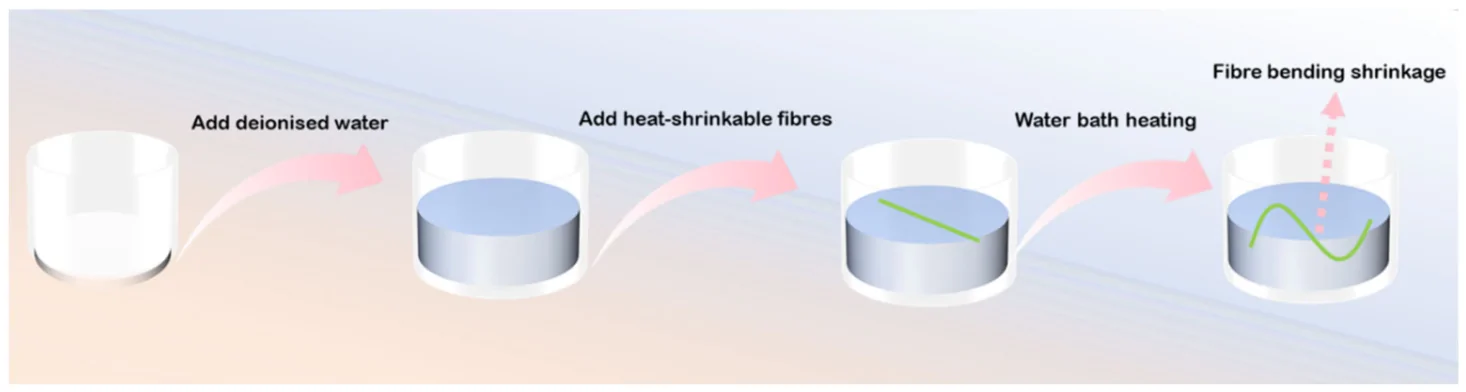
Flow chart for fiber heat shrinkage testing (the straight and curved green lines schematically denote the heat-shrinkable fiber before and after thermally activated shrinkage/bending in the water bath, respectively).

The free thermal shrinkage behavior of the HSFs was evaluated by measuring the axial length change during thermal exposure. Fiber specimens of predetermined initial lengths (*L*_1_ = 600, 800, or 1000 mm) were immersed in a constant-temperature deionized water bath maintained at preset temperatures (50, 60, 70, 80, or 90 °C). At designated time intervals, specimens were temporarily retrieved, and their instantaneous length (*L*_2_) was measured using a digital vernier caliper (precision: 0.01 mm). The free thermal shrinkage ratio (*φ*) was calculated according to Equation (1):

(1) $$\varphi =\frac{L_{1}-L_{2}}{L_{1}}\times 100\%$$

where *L*_1_ is the initial length before immersion (mm), and *L*_2_ is the length measured after immersion for a specified duration (mm). The overall testing procedure is illustrated in Figure 4. Three replicate specimens were tested for each group.

(3) Measurement of Embedded Fiber Strain and Calculation of Constrained Recovery Stress

**Figure 5:**
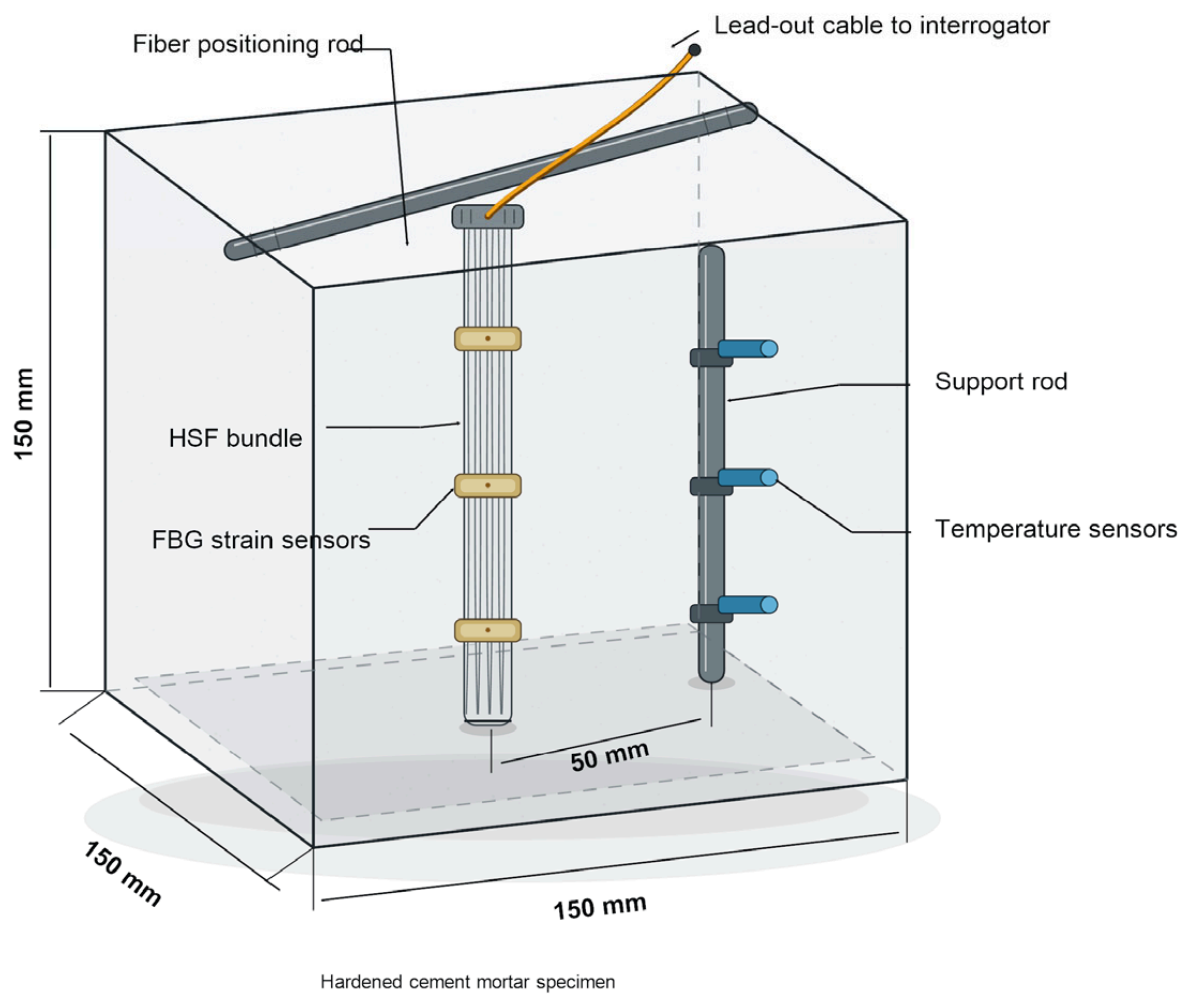
Schematic illustration of the embedded optical-fiber monitoring setup for measuring fiber recovery stress in mortar: configuration of the 150 mm cubic specimen, vertically positioned fiber bundle, optical fiber strain sensors, auxiliary positioning rod, and parallel rebar with temperature sensors (spacing: 50 mm; the grey shaded region denotes the cement mortar specimen, and the outer wireframe represents the cubic mold).

To evaluate the recovery stress that the fibers can develop within a cementitious matrix, an embedded optical-fiber strain sensing system was implemented in 150 × 150 × 150 mm cubic mortar specimens, as illustrated in Figure 5. The system records the axial strain of a continuous fiber bundle that is restrained by the hardening cement paste; the recovery stress is then calculated from this measured strain using Equation (2). No force was measured directly on the bundle, and the recovery stress reported in Section 3.1.3 is therefore a calculated rather than a directly measured quantity.

As shown in Figure 5, a continuous bundle of HSF monofilaments was vertically fixed and aligned along the central axis of the cubic mold using an auxiliary positioning rod. High-precision optical fiber Bragg grating (FBG) strain sensors—configured as a two-point grating array with a grating length of 5 mm, a center-to-center spacing of 50 mm, central reflection wavelengths of 1542 nm and 1545 nm, and 1.5 m pigtails at both ends—were affixed along the fiber bundle to monitor the axial strain ($\varepsilon_{f}$) induced by fiber contraction. Simultaneously, a separate calibrated thermistor (measurement range: −50 °C to 150 °C; measurement accuracy: ±0.5 °C; resolution: 0.1 °C; and average sensitivity: −2.1 mV/°C) was mounted on a parallel steel rebar at a horizontal distance of 50 mm to record the internal temperature history (*T*) and provide the temperature reference for compensating the strain-sensing FBG.

After casting with mortar, the specimen was cured at 20 ± 2 °C for 24 h to allow initial matrix hardening. The reference baseline condition ($\varepsilon_{f}=0$, baseline Bragg wavelength $\lambda_{0}$) was established at the end of this 24-h pre-curing period immediately before the heating cycle commenced. During heating, as the thermal activation triggered entropic recoiling of the molecular chains, the rigid surrounding paste prevented the fiber bundle from undergoing free longitudinal shrinkage. Consequently, an internal tensile elastic restraint strain was developed within the fiber core. To decouple the mechanical response from the temperature artifact, the thermal contribution has to be removed from the strain that the grating reports. Two corrections are possible, and only one of them may be used: either the temperature-induced shift is removed from the measured wavelength, or the temperature-induced strain is computed from the measured temperature history and subtracted. Applying both would remove the thermal contribution twice; the second was adopted here. First, the total apparent strain was recorded by the strain-sensing FBG as $\varepsilon_{total}\left(t\right)=\frac{\lambda_{B}\left(t\right)-\lambda_{B,0}}{\lambda_{B,0}(1-p_{e})}$, where $p_{e}$ is the photoelastic coefficient (~0.22). Second, the internal temperature history *T*(*t*) was monitored by the calibrated thermistor on the adjacent rebar, and the temperature-induced strain was determined as $\varepsilon_{T}(t)\;={\;K}_{T}[T(t)\;-\;T_{0}]$, where *K*_T_ is the combined temperature sensitivity coefficient of the FBG, and *T*_0_ is the baseline temperature. The manufacturer’s calibration data for the grating type used give this coefficient as $K_{T}\;=\;16.7\;\mu \varepsilon /{}^{\circ}C$, which was used directly for temperature compensation in this study; the calibration incorporates both the thermal-expansion and thermo-optic effects of the specific grating type used. Third, the net mechanical restraint strain was isolated by direct subtraction: $\varepsilon_{f}(T)=\varepsilon_{total}(T)-\varepsilon_{T}(T)$. The constrained recovery stress ($\sigma_{r}$) transferred from the fiber to the matrix at each target temperature (50, 60, 70, 80, and 90 °C) was calculated according to:

(2) $$\sigma_{r}=E_{f}\left(T\right)\cdot \varepsilon_{f}\left(T\right)$$

where $\varepsilon_{f}\left(T\right)$ is the dimensionless net mechanical strain measured by the embedded FBG after temperature compensation ($1\;\mu \varepsilon =10^{-6}$), and $E_{f}(T)$ is the room-temperature elastic modulus of fibers that had previously been exposed to the temperature T and then cooled to 23 °C (see Section 3.1.1). Equation (2) is therefore an elastic estimate of the recovery stress: the strain is measured, whereas the stiffness is an ex situ approximation of the in situ modulus at temperature T. The modulus decreases only slightly above 70 °C (11.5 GPa at 70 °C vs. 10.3 GPa at 90 °C), so the ex situ modulus approximation is acceptable within the scatter of the modulus measurements and is used mainly for ranking activation temperatures. Because the recovery stress was obtained from the measured strain, a single worked example is sufficient to make the calculation transparent: at the benchmark temperature of 70 °C the measured net mechanical strain was $\varepsilon_{f}=1190\;\mu \varepsilon \;(1.19\times 10^{-3})$. Using the modulus of 11.5 GPa (11,500 MPa), Equation (2) gives ${\sigma \;}_{r}=11,500\;MPa\times (1190\times 10^{-6})=13.7\;\mathrm{MPa}$. The same procedure was applied at the other activation temperatures.

(4) Curing Regimes

To investigate the performance of mortar under different thermal histories, two curing environments were implemented for hardened specimens [89,90]:

Standard Curing Regime (C and D series): After casting, specimens in molds were placed in a moist chamber at 20 ± 2 °C and ≥95% relative humidity (RH) for 24 h. After demolding, the specimens were immersed in water (or maintained in the standard curing room at 20 ± 2 °C and ≥95%RH) until testing at 7 and 28 days.

Steam-Curing Regime (ZC and ZD series): To simulate the thermal history of mass concrete and accelerate fiber activation, a steam-curing cycle was carried out in a programmable environmental chamber:

Pre-curing period: Molded specimens were stored at 20 ± 2 °C and ≥95%RH for 24 h before demolding.

Heating stage: After demolding, the temperature was ramped from 20 °C to 70 °C at a constant heating rate of 15 °C/h (duration: 3.33 h).

Constant temperature stage: Maintained isothermally at 70 ± 1 °C with a relative humidity exceeding 95% for 10 h.

Cooling stage: The chamber was cooled at a controlled rate of 12 °C/h down to ambient temperature (20 °C, duration: ~4.2 h).

Subsequent curing: Following the steam cycle, specimens for mechanical strength testing were transferred to standard curing (20 ± 2 °C, ≥95%RH) until 7 and 28 days, while specimens for drying shrinkage testing were transferred immediately to the drying room (20 ± 2 °C, 50 ± 4%RH).

(5) Workability and Mechanical Properties of Mortar

The workability (fluidity) of the fresh mortar was evaluated immediately after mixing in accordance with GB/T 2419-2021 [91] (Test Method for Fluidity of Cement Mortar) using a standardized flow table apparatus. The spread diameter of the mortar was measured in two perpendicular directions after 25 drops within 25 s, and the average value was reported as the mortar flow diameter.

The compressive and flexural strengths of hardened mortar specimens were determined at 7 and 28 days in accordance with GB/T 17671-2021 [92] (Test Method for Strength of Cement Mortar (ISO Method)). Prismatic specimens (40 × 40 × 160 mm) were tested for flexural strength using three-point loading (n = 3). The broken prism halves resulting from the flexural test were subsequently tested for compressive strength using a standard loading area of 40 × 40 mm (n = 6). The mean values and standard deviations were calculated for each batch.

(6) Drying Shrinkage Test

To simulate the internal temperature and humidity conditions of high-strength mass concrete, specimens were first subjected to the steam-curing regime prior to the drying shrinkage evaluation [68,89]. Prismatic mortar specimens (40 × 40 × 160 mm) with stainless-steel gauge studs embedded in both ends were prepared in accordance with the Chinese national standard JC/T 603-2004 [93] (Standard Test Method for Drying Shrinkage of Cement Mortar).

The specimens were demolded after 24 h of casting and immediately subjected to the steam-curing cycle described in Section 2.2 (4) [68,90]. Upon completion of steam curing and cooling to ambient temperature (20 ± 2 °C), the initial gauge length of the specimens (*L*_0_) was recorded using a digital length comparator (precision: 0.001 mm) as the reference baseline. Subsequently, the specimens were transferred to a constant-temperature and humidity drying room maintained at 20 ± 2 °C and 50 ± 4% relative humidity. The actual length reading (*L*_t_) was measured at drying ages of 1, 3, 7, 10, 14, 21, and 28 days. The drying shrinkage strain ($\varepsilon_{sh}$) at each age was calculated according to Equation (3):

(3) $$\varepsilon_{sh}=\frac{L_{0}-L_{t}}{L_{0}}\times 10^{6}\left(\mu \varepsilon \right)$$

where $\varepsilon_{sh}$ is the drying shrinkage strain of the mortar prism (με); *L*_0_ is the initial comparator reading recorded immediately after steam curing (mm); and *L*_t_ is the actual comparator reading measured at drying age *t* (mm). Three replicate prisms were tested for each batch (n = 3), and the average shrinkage strain was reported [68,94].

(7) Chemical, Thermal, and Crystallinity Characterization of Fibers

Fourier Transform Infrared (FTIR) Spectroscopy: The surface chemical functional groups of unmodified and modified fibers were characterized using a Fourier transform infrared spectrometer (Tensor 27, Bruker, Ettlingen, Germany) in Attenuated Total Reflectance (ATR) mode. Spectra were acquired across the wavenumber range of 4000 to 500 cm^−1^ with a spectral resolution of 4 cm^−1^ and an accumulation of 32 scans.

Thermogravimetric Analysis (TG/DTG): The thermal stability and decomposition behavior of the fibers were investigated using a thermogravimetric analyzer (STA 449 F3, Netzsch, Selb, Germany). Fiber samples weighing approximately 8.0 ± 0.5 mg were placed in alumina crucibles and heated from 30 °C to 800 °C at a constant heating rate of 10 °C/min under a continuous high-purity nitrogen purge (50 mL/min).

Differential Scanning Calorimetry (DSC) and Crystallinity Determination: The melting behavior and relative crystallinity of the HSFs following thermal activation at different temperatures (50, 60, 70, 80, and 90 °C for 30 min) were measured using a differential scanning calorimeter (Q2000, TA Instruments, New Castle, DE, USA). Specimens of approximately 6.0 ± 0.5 mg were sealed in standard aluminum pans and heated from 25 °C to 200 °C at a heating rate of 10 °C/min under a nitrogen flow of 50 mL/min. The relative crystallinity (*Xc*) was calculated using Equation (4):

(4) $$X_{c}=\frac{∆H_{m}}{\left(\omega_{PP}\cdot {∆H}_{m}^{0,PP}+\omega_{PE}\cdot ∆H_{m}^{0,PE}\right)}\times 100\%$$

where Δ*H*_m_ is the total experimental melting enthalpy determined by integrating the endothermic melting peak (J/g); $\omega_{PP}$ (0.80) and $\omega_{PE}$ (0.20) are the weight fractions of mPP and MHMWPE in the blend, respectively; and ${∆H}_{m}^{0,PP}$ (207.0 J/g) and $∆H_{m}^{0,PE}$ (293.0 J/g) represent the theoretical melting enthalpies of 100% crystalline polypropylene and polyethylene, respectively [95]. Three replicate runs were performed for each condition (n = 3).

## 3. Results and Discussion

### 3.1. Thermo-Mechanical Properties of Heat-Shrinkable Fibers

#### 3.1.1. Tensile Properties

**Figure 6:**
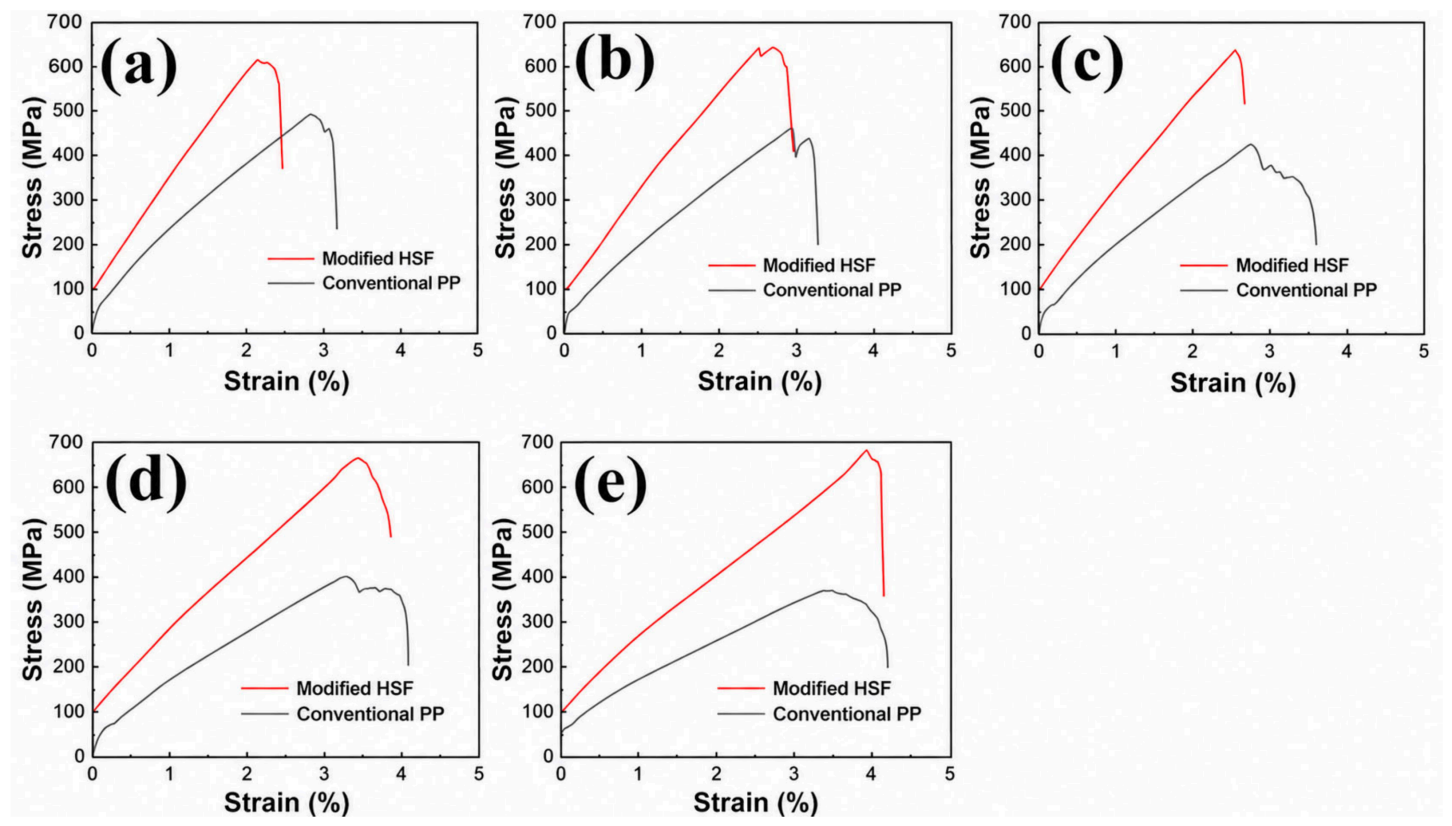
Room-temperature tensile stress-strain curves of fibers prepared at different hot-drawing temperatures: (**a**) 50 °C; (**b**) 60 °C; (**c**) 70 °C; (**d**) 80 °C; (**e**) 90 °C.

As shown in Figure 6, the hot-drawing temperature applied during fiber fabrication exerts a significant influence on the room-temperature tensile behavior of the heat-shrinkable fibers (HSFs). All tensile tests were conducted at ambient temperature (23 ± 2 °C) on fiber specimens prepared at drawing temperatures ranging from 50 °C to 90 °C. With the drawing temperature increasing from 50 °C to 90 °C, both the room-temperature tensile strength and ultimate tensile strain of the modified HSFs exhibit a gradual and synchronous improvement. Specifically, for fibers drawn at 50 °C (Figure 6a), the tensile strength is approximately 602 ± 18 MPa with an ultimate strain of around 3.5 ± 0.3%. When the drawing temperature is increased to 90 °C (Figure 6e), the tensile strength reaches nearly 682 ± 22 MPa, accompanied by an ultimate strain of approximately 4.8 ± 0.4%. This concurrent enhancement in both strength and ductility indicates that higher drawing temperatures facilitate more uniform molecular chain alignment along the drawing axis while minimizing internal micro-defects.

**Figure 7:**
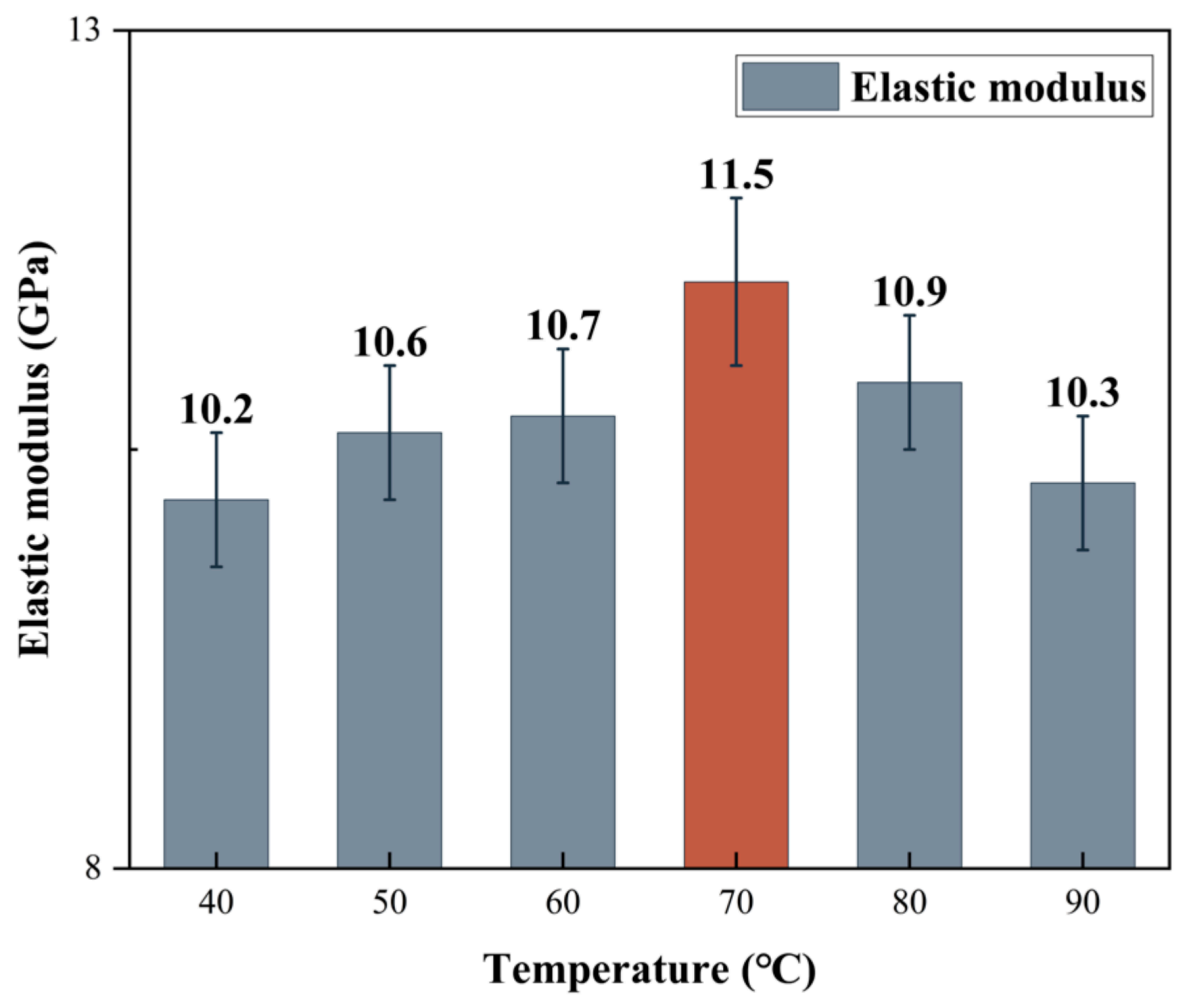
Room-temperature elastic modulus of heat-shrinkable fibers after thermal activation at different temperatures (the red bar highlights the maximum modulus achieved at the benchmark activation temperature of 70 °C; fibers exposed to target temperatures for 30 min, cooled to 23 °C, and tested at room temperature; error bars represent ±1 standard deviation, n = 5).

In comparison, the conventional PP control fibers tested under identical conditions exhibited significantly lower peak tensile strengths, typically in the range of 360–470 MPa across the drawing temperatures (Figure 6), which aligns with the typical performance range reported for unmodified polyolefin fibers (300–450 MPa) [39,41]. The superior tensile performance of the modified HSFs stems from a powerful synergy between molecular orientation and chemical modification. The elevated hot-stretching temperature enhances polymer chain mobility, enabling them to overcome physical entanglements and align highly along the drawing axis. This alignment effectively directs the covalent bonds of the polymer backbone along the loading direction, drastically boosting axial load-bearing capacity. Critically, the radiation-induced crosslinked network provides a stable chemical skeleton. This structure prevents premature chain slippage and limits excessive molecular relaxation during hot drawing, leading to more uniform stress distribution among the polymer chains. Consequently, internal micro-defects are suppressed, resulting in the observed simultaneous improvements in tensile strength and deformation capacity. In addition to tensile strength, the elastic modulus is another key parameter governing the reinforcing efficiency of HSFs in cementitious composites. To evaluate the effect of thermal exposure on stiffness retention, fiber specimens were thermally activated by immersion in a constant-temperature water bath at designated temperatures (40–90 °C) for 30 min in an unrestrained state, followed by cooling at ambient temperature (23 °C) for 2 h. Their elastic moduli were subsequently measured at room temperature. Figure 7 presents the variation in the room-temperature elastic modulus of the HSFs as a function of activation temperature. Unlike the monotonic increase in tensile strength, the elastic modulus exhibits an inverted U-shaped trend. Specifically, the modulus increases from 10.2 ± 0.4 GPa at 40 °C to a peak of 11.5 ± 0.5 GPa at 70 °C, followed by a slight decrease to 10.3 ± 0.4 GPa at 90 °C. The initial increase in elastic modulus can be attributed to enhanced molecular rearrangement and secondary crystallization during moderate thermal activation. As the activation temperature increases from 40 to 70 °C, the mobility of the polymer chains promotes the formation of a more ordered crystalline structure while reducing structural imperfections within the fibers, thereby improving their stiffness. However, when the activation temperature exceeds 70 °C, excessive molecular mobility may induce the relaxation or partial reorganization of less stable crystalline regions, resulting in a slight reduction in crystallinity and, consequently, elastic modulus.

Despite this modest decrease at higher activation temperatures, the HSFs retain an elastic modulus above 10.2 GPa across the entire temperature range. This stable stiffness is critical. A higher modulus favours the transfer of the thermally generated recovery stress to the surrounding matrix. When paired with the contraction of the HSFs, this stress transfer contributes to internal compressive restraint. This restraint actively counteracts the tensile stresses arising during hydration and subsequent cooling, thereby enhancing the overall crack resistance of high-strength cement mortar.

#### 3.1.2. Thermal Shrinkage Behavior

(1) Effect of Fiber Length on Thermal Shrinkage Ratio

**Figure 8:**
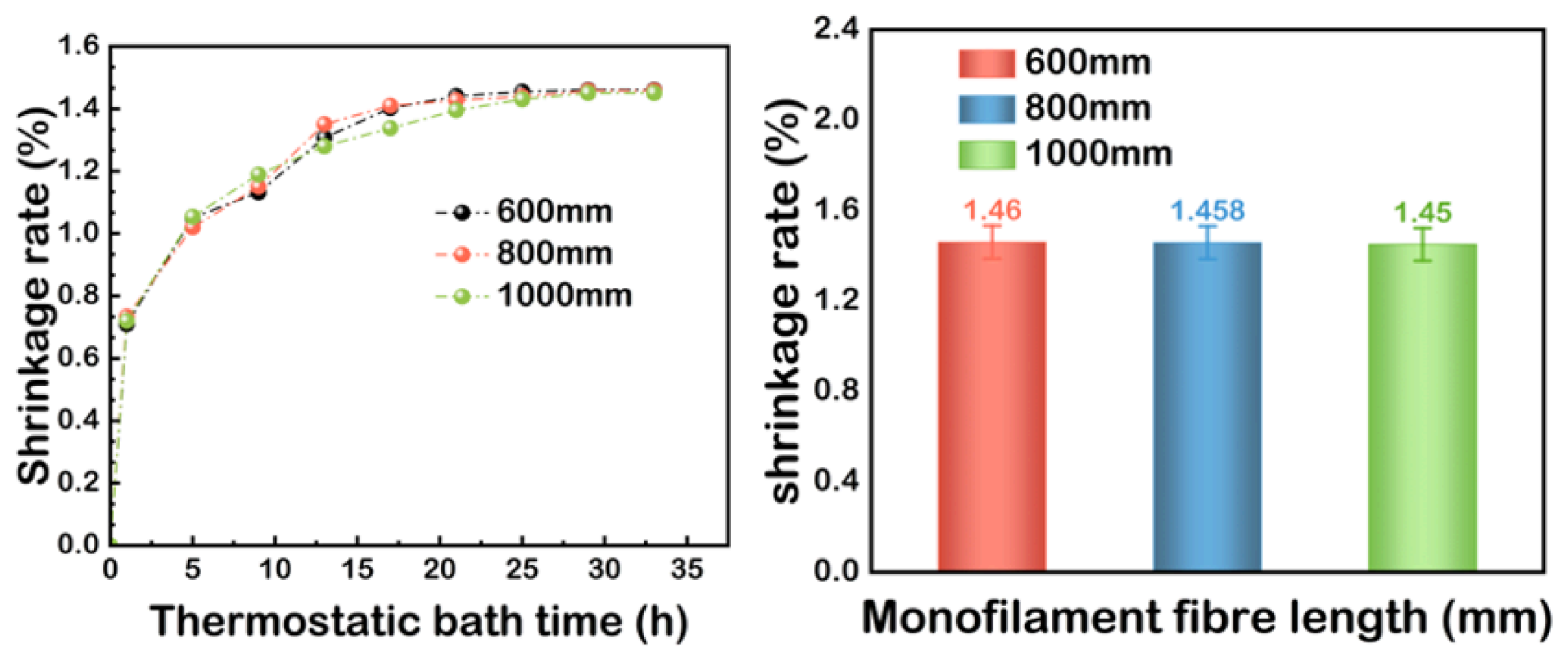
Variation of thermal shrinkage ratio of heat-shrinkable fibers with different lengths at 70 °C (n = 3; error bars represent ±1 standard deviation).

Figure 8 shows the evolution of the thermal shrinkage ratio of HSFs with different initial lengths during heating at 70 °C. For all fiber lengths, the shrinkage ratio increases rapidly during the initial stage of heating and gradually approaches a stable value with prolonged heating time. The shrinkage curves almost completely overlap, indicating that the initial fiber length has a negligible influence on the thermal shrinkage ratio.

The rapid increase in shrinkage at the early stage is attributed to the thermally activated relaxation of the highly oriented polymer chains generated during the drawing process. As the activation proceeds, the stored orientation strain is progressively released, resulting in axial contraction of the fibers. With increasing heating time, the number of recoverable oriented molecular segments gradually decreases, leading to a reduction in the shrinkage rate until a stable state is reached.

The negligible effect of fiber length indicates that the thermal shrinkage ratio is governed primarily by the intrinsic shape-memory characteristics of the polymer rather than by specimen geometry. Under identical thermal conditions, fibers with different initial lengths undergo a similar degree of molecular relaxation and release a comparable proportion of the stored orientation strain, thereby exhibiting nearly identical shrinkage ratios. This result shows that the thermally induced shrinkage behavior of the HSFs is highly stable and reproducible over a wide range of fiber lengths, which is advantageous for practical applications in cementitious materials where fibers are randomly distributed with varying embedded lengths.

(2) Effect of Activation Temperature on Thermal Shrinkage Ratio

**Figure 9:**
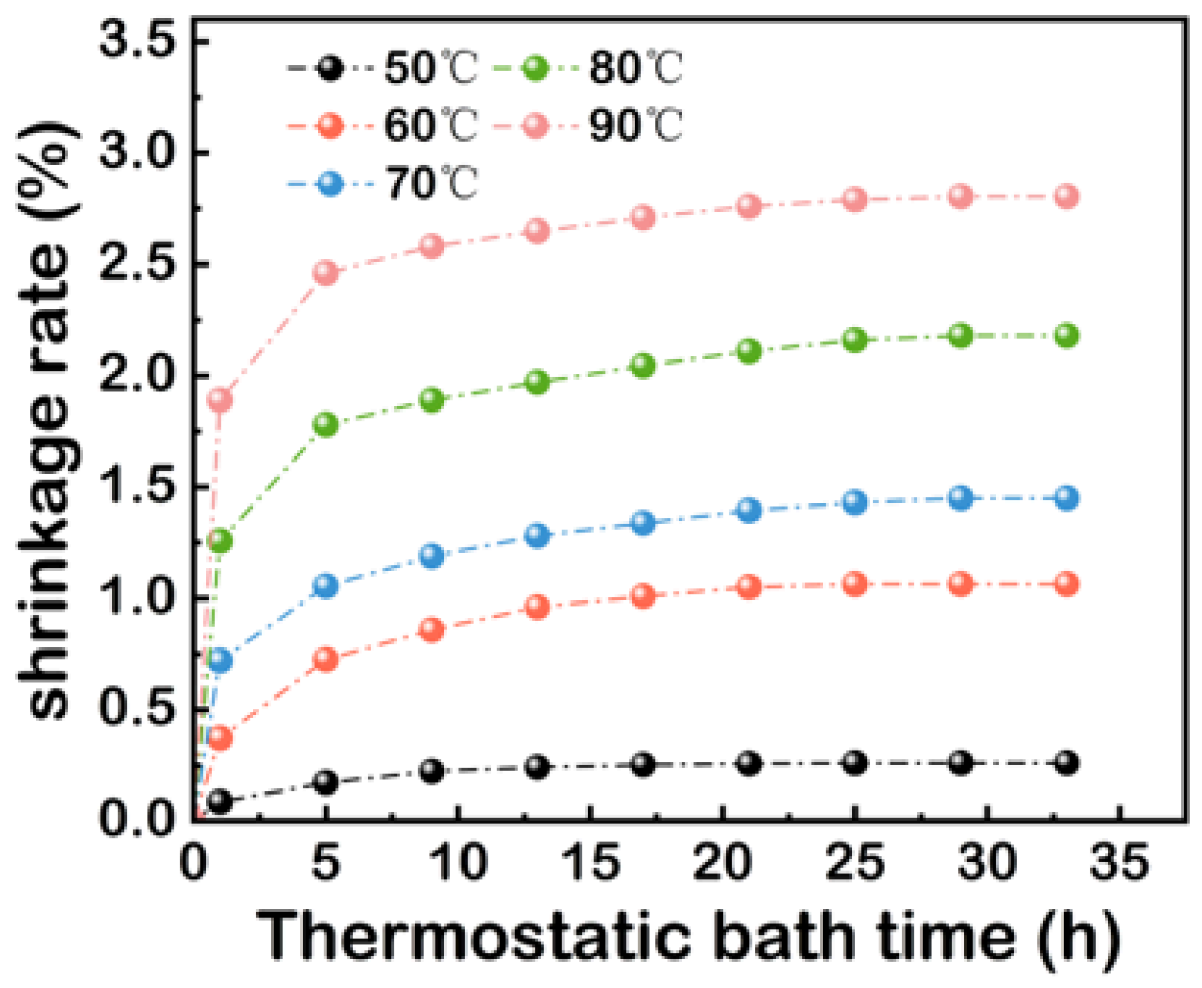
Time-dependent thermal shrinkage curves of heat-shrinkable fibers at different temperatures (n = 3).

Figure 9 shows the evolution of the thermal shrinkage ratio of HSFs (initial length: 1000 mm) under different water-bath temperatures. For all temperatures, the shrinkage ratio increases rapidly during the initial stage of heating and gradually approaches a stable value with prolonged heating time, indicating the progressive completion of the thermally activated recovery process.

The activation temperature markedly influences both the shrinkage rate and the ultimate shrinkage ratio. As the temperature increases from 50 to 90 °C, the final shrinkage ratio increases from approximately 0.26% to 2.80%. Moreover, higher temperatures significantly accelerate the shrinkage process. At 90 °C, most of the total shrinkage occurs within the first hour of heating, whereas only limited shrinkage is observed at 50 °C over the same period. These results indicate that increasing the activation temperature not only accelerates the recovery kinetics but also promotes a greater release of the stored orientation strain.

This temperature-dependent behavior originates from the shape-memory characteristics of the modified polyolefin fibers. At relatively low temperatures, molecular mobility remains limited, and only a small proportion of the oriented polymer chains is able to relax. As the activation temperature increases, enhanced chain mobility facilitates the progressive relaxation of the highly oriented molecular structure, resulting in greater axial contraction of the fibers. As evidenced by the plateau behavior in Figure 9, this thermally induced shrinkage stabilizes rapidly; owing to the permanent radiation-crosslinked network and the irreversible nature of entropic chain recoiling, the contracted configuration is effectively locked in, allowing the activated fibers to maintain a stable contracted state without rebound upon subsequent cooling. This stable shrinkage behavior provides the basis for generating thermal recovery stress in cementitious materials, thereby contributing to internal compressive restraint and improved crack resistance during hydration and subsequent cooling.

The thermal shrinkage characteristics of the developed HSFs—the onset shrinkage temperature and the ultimate shrinkage ratio—are tunable rather than fixed. These parameters can be calibrated by adjusting the molecular weight and volume fraction of the PE phase, regulating the electron-beam radiation dose to tailor the crosslinking density (consistent with the crystallinity and modulus evolution discussed in Section 4.4), and optimizing the draw ratio during the post-drawing fibrillation stage. This exceptional customizability allows for a ‘targeted’ or bespoke thermal prestressing design tailored to mass concrete components with varying structural thicknesses and distinct hydration heat curves. Consequently, the HSF system can be engineered to trigger contraction in tandem with the specific thermal profile of a given concrete structure, providing a highly adaptive and proactive strategy for early-age crack mitigation in diverse civil engineering applications.

From a macromolecular physics perspective, the heating kinetics (i.e., heating rate, dT/dt) play a decisive role in governing the thermo-mechanical response of semi-crystalline polymer blends by regulating the competitive kinetics between two concurrent mechanisms: entropic chain relaxation and secondary crystallization.

#### 3.1.3. Calculated Constrained Thermal Recovery Stress

**Figure 10:**
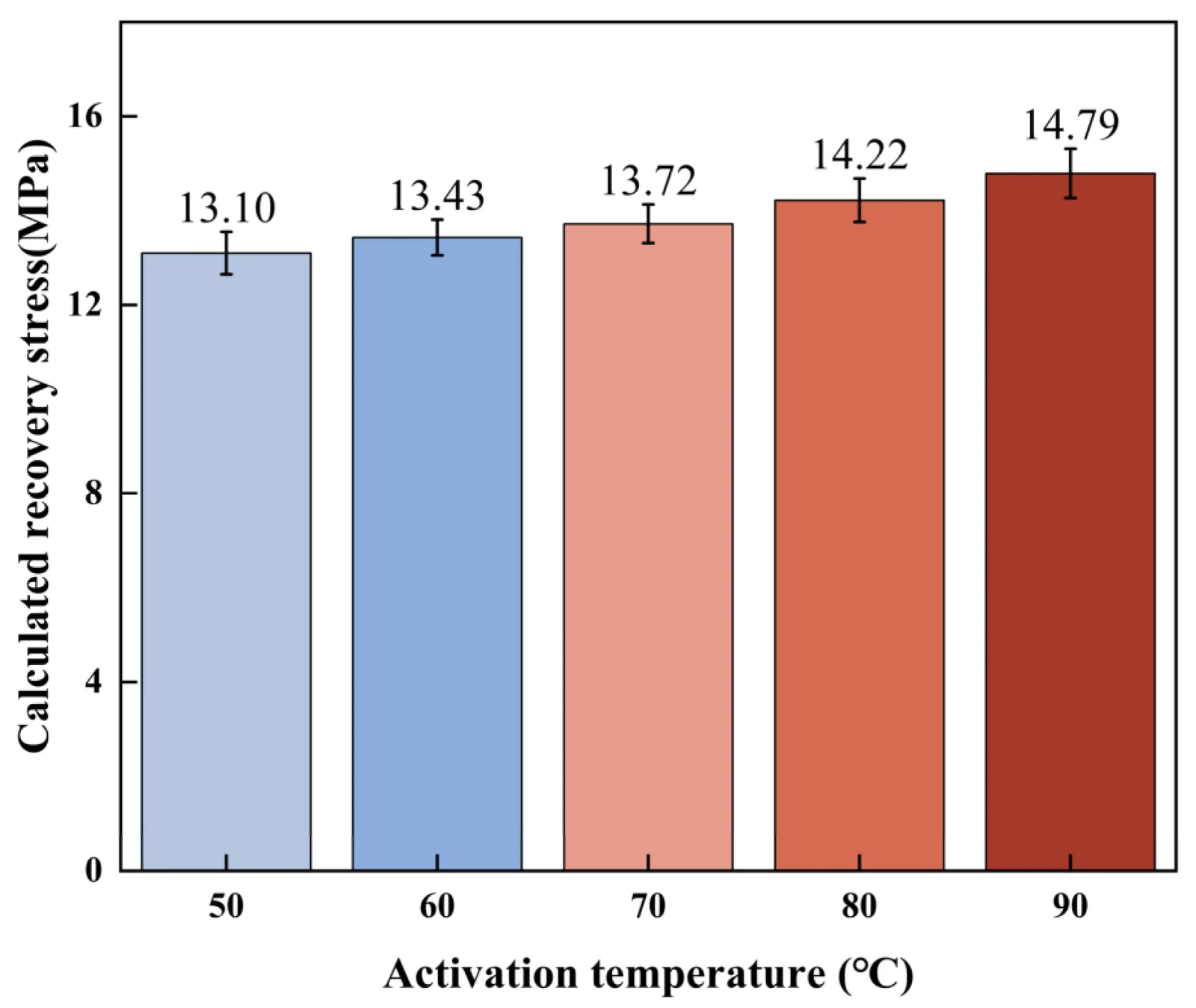
Calculated constrained thermal recovery stress of the HSFs at different activation temperatures ($\sigma_{r}=\;E_{f}\cdot \varepsilon_{f}$, where $\varepsilon_{f}$ is the net mechanical strain measured by the embedded FBG and *E*_f_ is the modulus reported in Figure 7; error bars represent ±1 standard deviation, n = 3).

Figure 10 presents the constrained thermal recovery stress of the HSFs at various activation temperatures, calculated from the measured FBG strains using Equation (2). The calculated stress increases monotonically with temperature, from 13.10 ± 0.45 MPa at 50 °C to 14.79 ± 0.52 MPa at 90 °C. Even at the onset temperature of 50 °C, the restrained fibers develop a calculated stress above 13 MPa, indicating a high thermal sensitivity and a rapid initiation of prestressing. Because the strain input is measured on a continuous, unidirectionally aligned bundle, these values describe the recovery stress that the fiber material can generate under ideal one-dimensional restraint; they do not represent the stress state of the surrounding matrix.

These values represent the axial tensile stress developed within the fiber bundle under the rigid constraint of the surrounding cement mortar, obtained from the strain measured by the embedded sensing system (Figure 5) and converted with Equation (2). Upon thermal activation, the oriented molecular chains tend to recoil, leading to unconstrained shrinkage if unrestrained. However, when embedded within the hardening cementitious matrix, this contraction is firmly restricted. Consequently, the actual shrinkage strain of the embedded fibers is much smaller than their free shrinkage capacity (2.80% at 90 °C), resulting in the buildup of a sustained axial tensile stress of 13–15 MPa inside the fiber core. This axial stress is the quantity that would drive pre-compression of the surrounding paste if the bond were perfect; in the composite it is reduced by the factors discussed below. It is essential to distinguish the boundary conditions of this measurement from the actual behavior of short fibers in composite mortar. The embedded FBG setup utilizes a continuous, unidirectionally aligned fiber bundle with continuous matrix gripping, representing the idealized, upper-bound recovery stress capacity of the HSF material under 1D rigid constraint. In contrast, the 12 mm discrete fibers randomly dispersed in the actual mortar are subject to 3D spatial orientation efficiency, finite embedment length, and interfacial shear slip, which will naturally reduce the macroscopic effective stress transfer compared to this continuous bundle benchmark.

Through interfacial shear stress transfer, this inward tensile recovery stress developed in the activated fibers is transmitted to the adjacent cementitious paste. Under restrained conditions, the entropic contraction of the embedded HSFs generates shear traction along the fiber–matrix interface during early hydration hardening. Unlike conventional passive fibers that only mobilize bridging forces after matrix cracking has already occurred, this proactive interfacial stress transfer exerts an inward confining traction on the surrounding paste. This mechanism can qualitatively contribute to localized pre-compressive effects around the fibers, thereby counteracting early capillary tensile stress accumulation and reducing the driving force for early microcrack initiation.

However, because the actual stress distribution inside the composite depends on several interacting factors—such as the evolving viscoelastic modulus of the hydrating paste, dynamic interfacial bond-slip behavior, and the 3D random spatial orientation of the short fibers—a simplified analytical conversion from fiber axial recovery stress to bulk matrix pre-compression cannot be rigorously justified without extensive multi-scale numerical calibration. Therefore, the calculated recovery stress (13.72 MPa at 70 °C) is interpreted strictly as the intrinsic driving potential of the fibers, and direct quantification of the internal matrix stress field remains an objective for future micro-mechanical work. It is neither a measurement of compressive stress in the matrix nor evidence that such compression develops; its role in this study is limited to ranking the activation temperatures and to supporting a qualitative mechanism.

Although the recovery stress reaches its maximum at 90 °C (14.79 MPa), the value developed at 70 °C is already 13.72 MPa, i.e., 92.8% of the peak, and the elastic modulus peaks at that same temperature (11.5 GPa, Section 3.1.1). The choice of 70 °C is therefore governed by the thermo-mechanical balance of the fiber system rather than by the maximum stress alone. At 70 °C, secondary crystallization is the most stable and gives the fiber its highest stiffness while it mobilizes nearly the whole of its recovery capacity, whereas temperatures up to 90 °C raise the stress further (14.79 MPa) at the cost of relaxing the less stable crystalline regions and lowering the modulus to 10.3 GPa, without harmful degradation. In addition, 70 °C coincides with the core temperature plateau typically developed during the hydration of mass concrete (60–80 °C). It was therefore adopted as the benchmark activation temperature for the mortar tests, where stress generation, load-transfer stiffness and a realistic thermal history are best combined.

Kinetic Compatibility with Precast Steam Curing: In modern precast concrete manufacturing, standard steam-curing regimes typically comprise a controlled heating rate of 10–15 °C/h, an isothermal holding stage at 60–75 °C for 6–10 h, and controlled cooling. As revealed by the time-dependent shrinkage curves in Figure 9, the entropic recovery kinetics of the HSFs are exceptionally fast: at 70 °C, approximately 70% of the ultimate shrinkage is mobilized within the first 2 h (and over 85% within 6 h), with full plateau contraction essentially achieved within the 6–10 h isothermal stage. Peak holding times typical of industrial practice (6–10 h) are therefore long enough for the shape-memory recovery to develop. Furthermore, the moderate industrial heating rate (10–15 °C/h) avoids premature thermal shocking, allowing the cementitious matrix to achieve initial set and stiffness prior to significant fiber contraction, which favours efficient shear-stress transfer along the interface.

In-Situ Thermal Activation in Mass Concrete: In massive structural pours (such as raft foundations, bridge pylons, and dam blocks), exothermic cement hydration under semi-adiabatic conditions naturally drives internal temperatures into the range of 60–85 °C within 36–72 h. Because the HSF exhibits an onset activation temperature of T_onset_ = 50 °C, the massive internal hydration heat provides sufficient thermal energy to trigger in-situ fiber contraction without requiring any external energy input. Crucially, the fiber’s thermal contraction is initiated during the heating stage, and the resultant recovery stress is locked into the hardening matrix. When the structure subsequently enters the cooling and drying phase (when thermal contraction tensile stresses accumulate and provoke cracking), this internally locked-in pre-compression acts as an active counteracting stress, effectively mitigating early-age crack initiation.

Although the present evaluation was conducted at the laboratory mortar scale, extending these findings to structural concrete involves several favorable multi-scale compatibilities. Because synthetic fibers reside exclusively within the paste fraction (which typically accounts for 55–65% of the total concrete volume), the identified optimal mortar dosage ($V_{f,mortar}=0.3\%$) directly scales to a whole-concrete volume fraction of approximately 0.18–0.20% (about 1.7–1.9 kg/m^3^). Furthermore, the 12 mm fiber length maintains a favorable geometric ratio with typical coarse aggregate sizes (d_max_ = 16–20 mm, with L_f_/d_max_ ≤ 0.75), preventing the aggregate wall-effect and ensuring smooth inter-particle dispersion during mixing. In solid concrete, rigid coarse aggregates act as non-shrinking inclusions that induce severe localized tensile stress concentrations in the surrounding paste; here, the thermally activated recovery stress (13.72–14.79 MPa) is positioned precisely to reinforce these vulnerable aggregate–paste interfacial transition zones. Concurrently, the 50–90 °C core hydration temperature naturally developed in mass concrete matches the fiber’s 50–90 °C activation window (T_onset_ = 50 °C), enabling autonomous in-situ contraction without external heating. Although validating the stress-compensation efficiency under multi-axial structural restraint remains to be addressed in future work, these analyses indicate that the mortar-scale mechanism is, in principle, transferable to structural mass concrete; verification at that scale remains outstanding.

### 3.2. Effect of HSFs on the Properties of Cement Mortar

The reinforcing performance of heat-shrinkable fibers (HSFs) in cement mortar is influenced primarily by their geometric characteristics and dosage. Fiber length determines the dispersion, crack-bridging efficiency, and activation effectiveness of the HSFs within the mortar matrix, whereas fiber dosage governs the number of reinforcing fibers participating in crack control. Therefore, this section investigates the influence of fiber length and dosage on the workability, mechanical properties, and volumetric stability of mortar to identify the optimum incorporation parameters.

#### 3.2.1. Effect of Fiber Length

The measured apparent density of the modified HSFs is 0.93 g/cm^3^. In this series the fiber volume fraction was held constant at 0.3%, so the fiber mass was the same in every mix (1.89 g per batch) and only the fiber length was varied (8, 12, 16 and 20 mm). The admixture dosage refers to the as-delivered commercial PCE solution.

Based on the thermal response characteristics presented in Section 3.1, HSFs with lengths of 8, 12, 16, and 20 mm were selected to investigate the influence of fiber length on mortar properties. The water-to-binder ratio was maintained at 0.30. The corresponding specimens were designated as C1–C4 under standard curing and ZC1–ZC4 under steam curing (peak temperature of 70 °C). The mix proportions are listed in Table 3.

**Figure 11:**
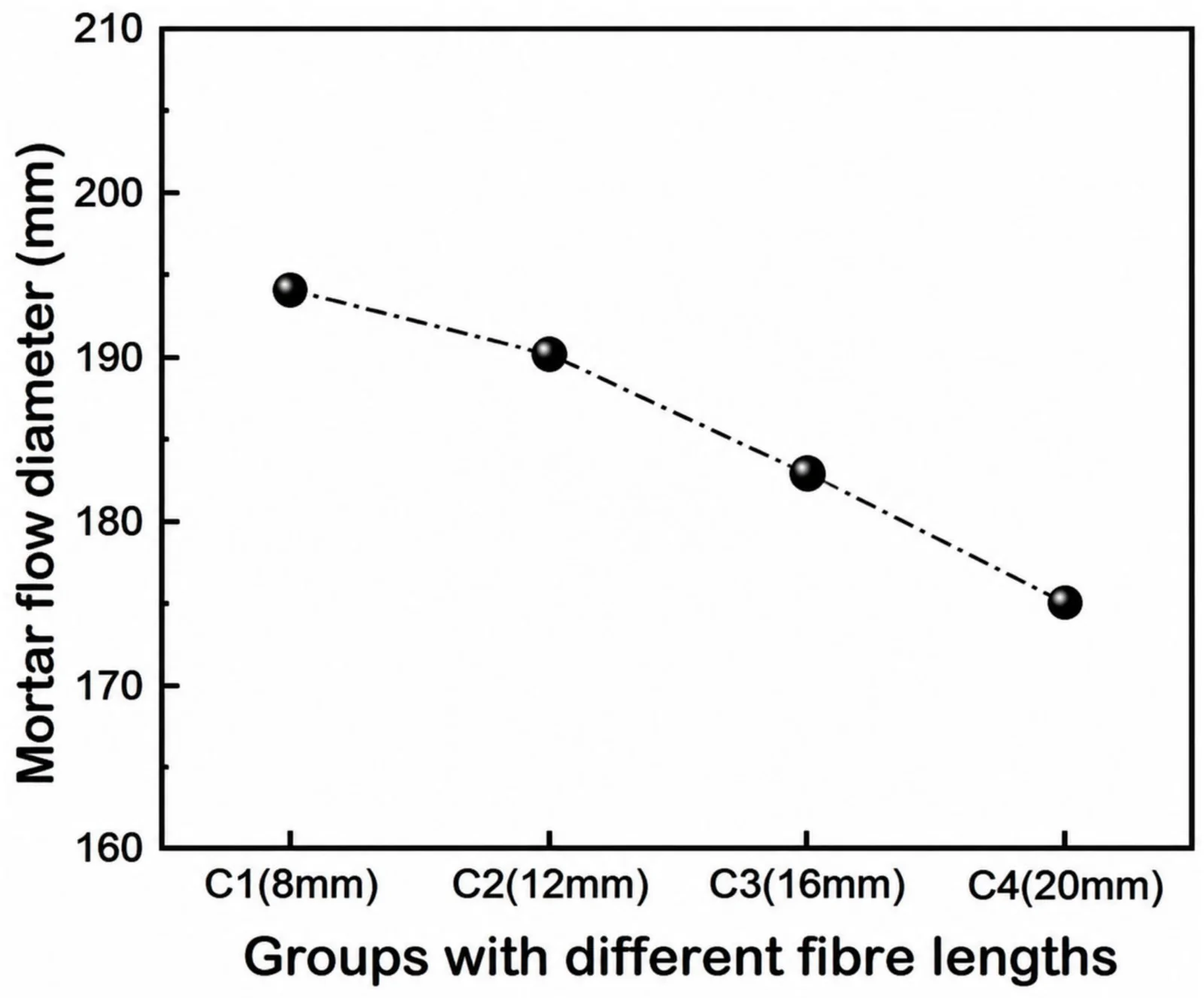
Mortar flow diameter with different heat-shrinkable fiber lengths (measured across two perpendicular diameters per batch per GB/T 2419).

As shown in Figure 11, the flowability of the mortar decreases gradually with increasing fiber length. Longer fibers are more likely to form interconnected networks and increase mechanical interlocking within the fresh mortar, thereby increasing the resistance to flow. In addition, longer fibers exhibit a greater tendency to overlap and entangle, resulting in reduced dispersion and increased paste viscosity. Consequently, excessive fiber length adversely affects workability and may reduce the uniformity of fiber distribution.

**Figure 12:**
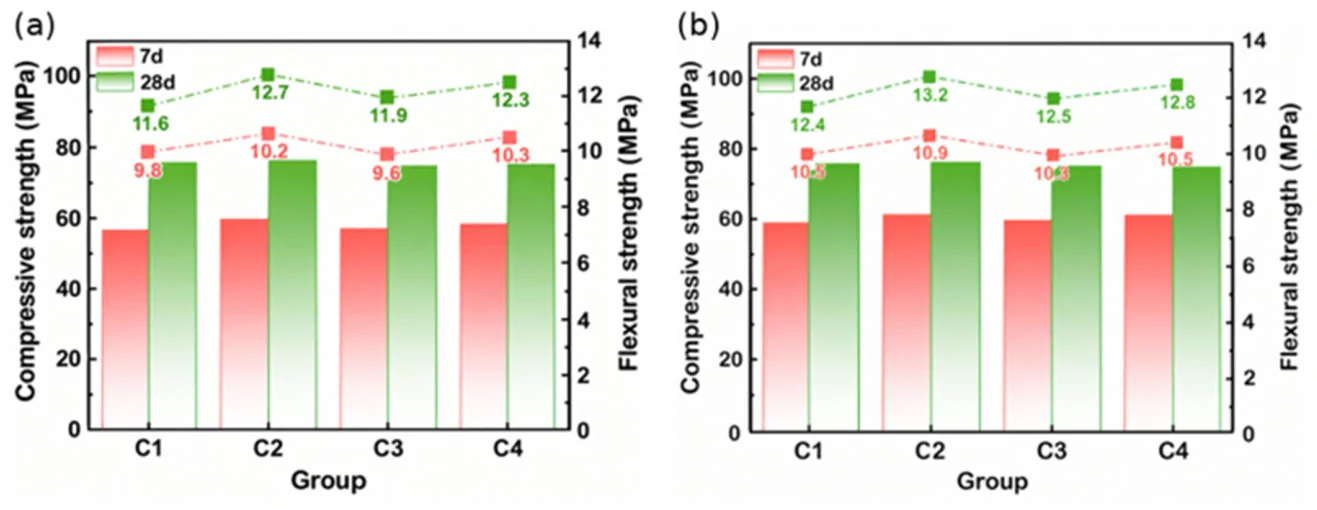
Compressive and flexural strengths of cement mortar with different fiber lengths: (**a**) Standard curing; (**b**) Steam curing (columns denote compressive strength; red and green dashed lines represent 7-day and 28-day flexural strengths, respectively; flexural strength: n = 3; compressive strength: n = 6).

Figure 12 shows that variations in fiber length (8 to 20 mm) produce minor changes in compressive strength (remaining within 75.5 ± 1.8 to 76.8 ± 2.1 MPa for standard curing, and 76.0 ± 2.0 to 77.8 ± 2.2 MPa for steam curing). In contrast, flexural strength shows a measurable dependency on fiber length, reaching a peak of 12.7 ± 0.4 MPa (standard curing) and 13.2 ± 0.5 MPa (steam curing) at 12 mm.

**Figure 13:**
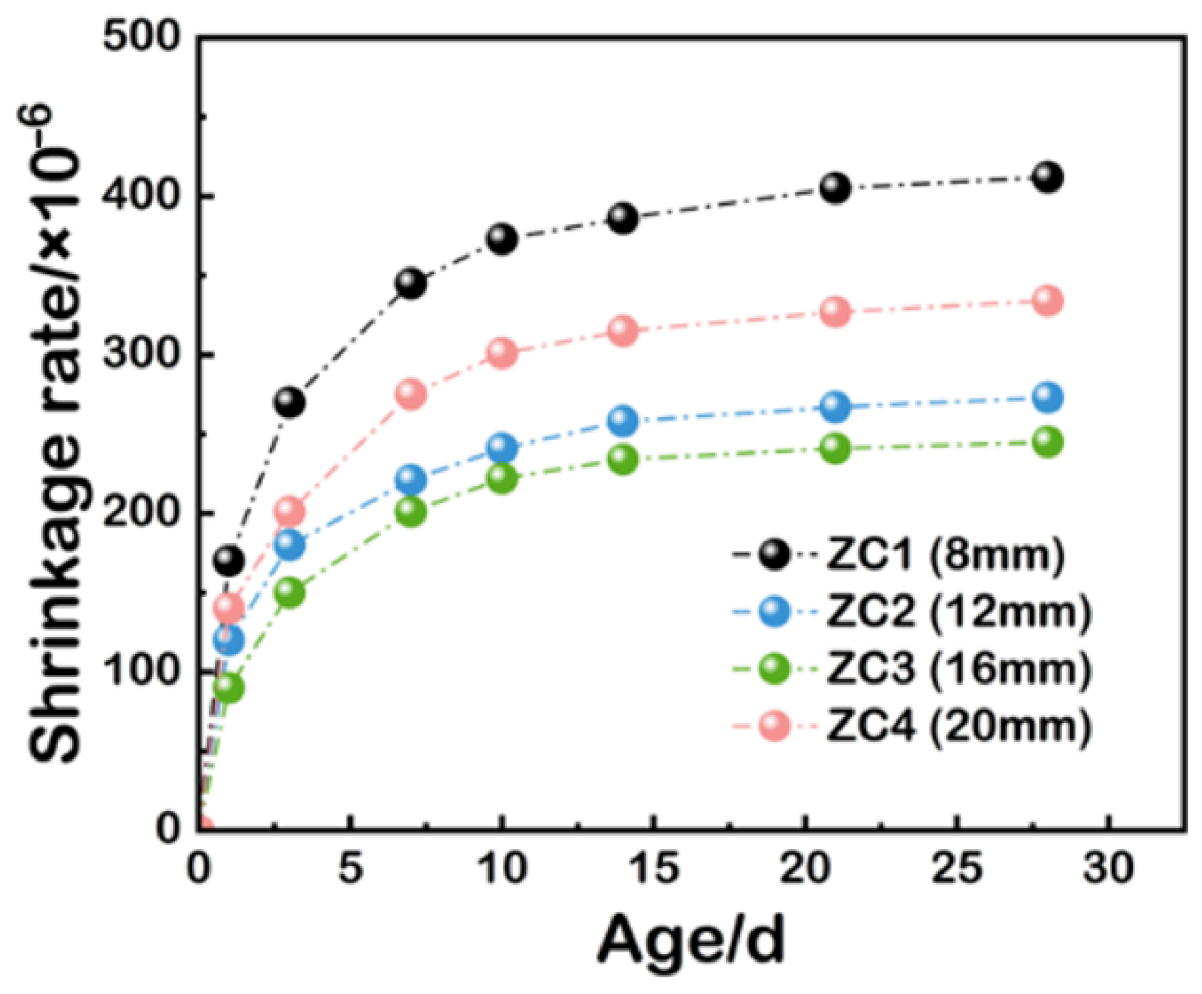
Drying shrinkage evolution of steam-cured mortar specimens with different fiber lengths (ZC series, 70 °C steam curing; baseline t = 0 recorded immediately after cooling to 20 °C; n = 3).

The influence of fiber length on drying shrinkage under Steam Curing Regime (ZC series) is presented in Figure 13. Mortars reinforced with 12–16 mm HSFs exhibit the lowest drying shrinkage, indicating that this fiber length range provides the most effective restraint against shrinkage deformation. Fibers of appropriate length are more uniformly distributed throughout the mortar and establish an effective three-dimensional reinforcing network that restrains the initiation and propagation of microcracks during moisture loss. In contrast, the reinforcing effect of 8 mm fibers is limited by their relatively short embedment length and reduced crack-bridging capability. When the fiber length is increased to 20 mm, the shrinkage-restraining efficiency slightly diminishes, which is likely attributable to less uniform spatial dispersion and potential fiber clustering, though direct dispersion quantification was not performed.

It is noteworthy that the superiority of the 12–16 mm HSFs is consistently reflected in both the mechanical properties and shrinkage resistance. This indicates that an appropriate fiber length keeps workability and dispersion acceptable and allows the activated HSFs to transfer recovery stress to the surrounding cementitious matrix during steam curing, thereby enhancing crack resistance while maintaining the overall integrity of the mortar.

#### 3.2.2. Effect of Fiber Dosage

Based on the results presented in Section 3.2.1, HSFs with a length of 12 mm exhibited the most favorable overall performance and were therefore selected for the dosage study. The fiber volume fractions were 0%, 0.1%, 0.3%, 0.5%, and 0.7%, corresponding to groups D1–D5, respectively. The corresponding steam-cured specimens (ZD1–ZD5) were subjected to the steam-curing regime (peak temperature of 70 °C) to simulate the thermal history of mass concrete. The mix proportions are listed in Table 4.

**Figure 14:**
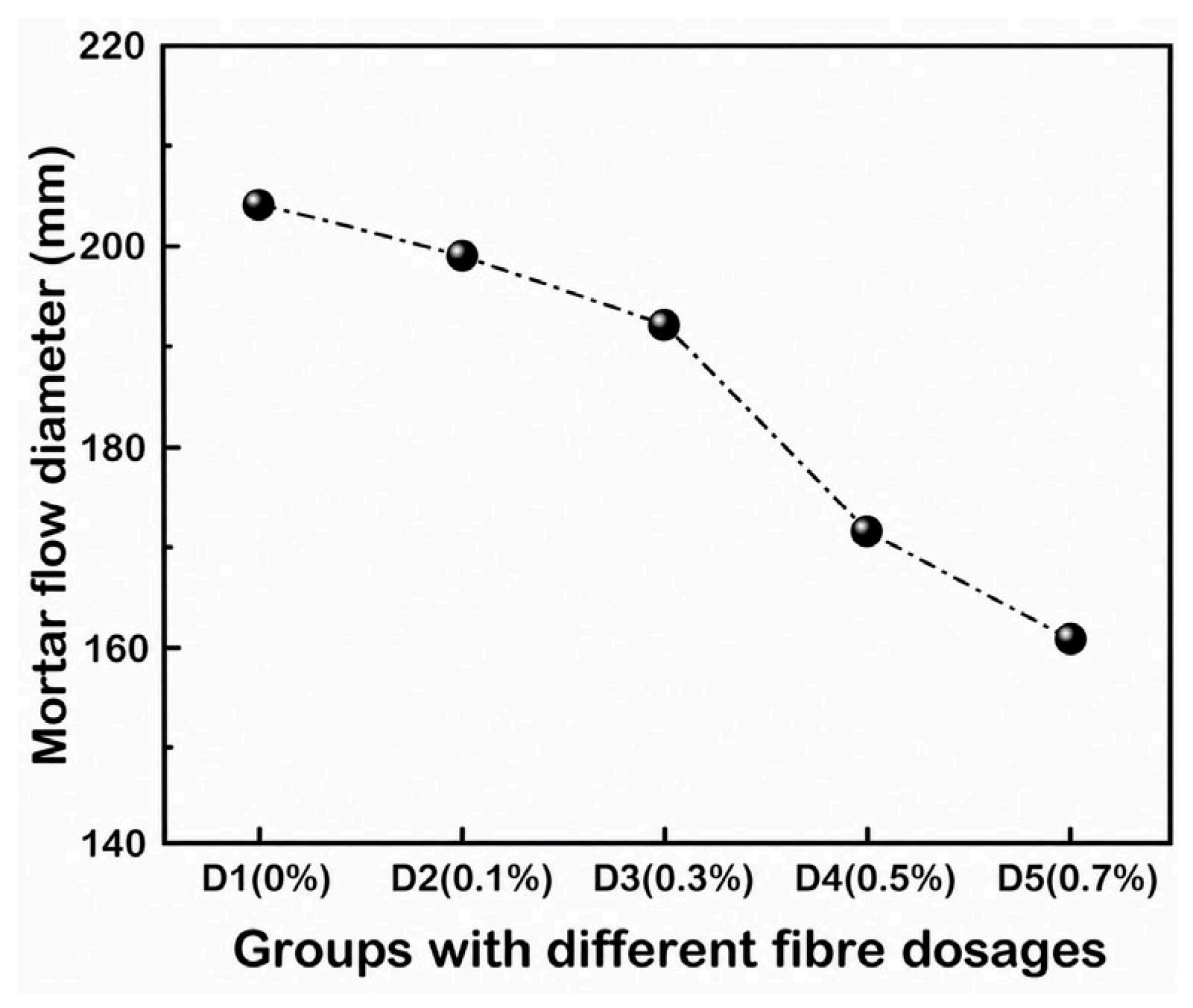
Mortar flow diameter with different heat-shrinkable fiber dosages (measured across two perpendicular diameters per batch per GB/T 2419).

Figure 14 shows that the flowability of the mortar decreases continuously with increasing fiber dosage. The reduction becomes more pronounced when the fiber volume fraction exceeds 0.3%. Increasing the fiber content enlarges the specific surface area requiring paste coating and enhances mechanical interlocking among fibers, thereby increasing the viscosity and flow resistance of the fresh mortar. At relatively high dosages (0.5% and 0.7%), a significantly increased fiber count increases inter-fiber contact probability, which can heighten internal friction and flow resistance within the fresh paste, further lowering fluidity.

**Figure 15:**
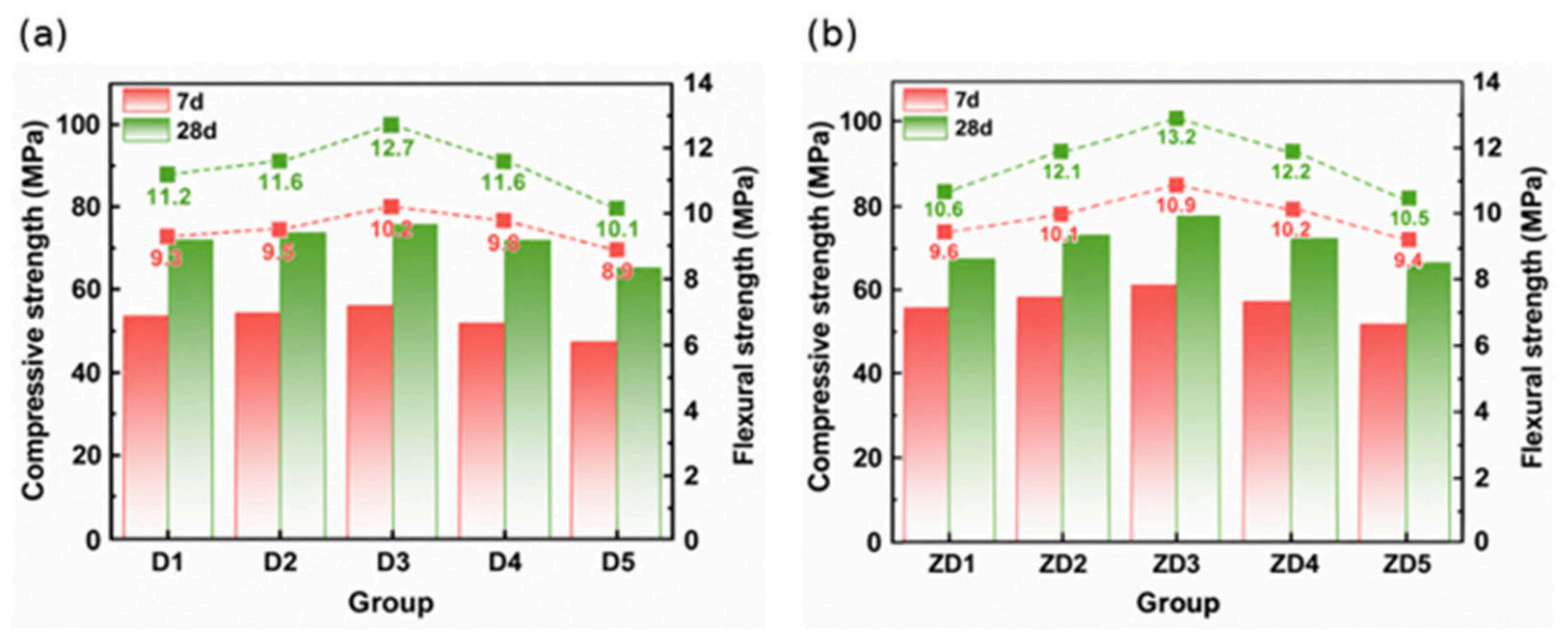
Compressive and flexural strengths of cement mortar with different fiber dosages: (**a**) Standard curing; (**b**) Steam curing (columns denote compressive strength; red and green dashed lines represent 7-day and 28-day flexural strengths, respectively; flexural strength: n = 3; compressive strength: n = 6).

The influence of fiber dosage on the mechanical properties is presented in Figure 15. Both the compressive and flexural strengths initially increase and then decrease with increasing fiber dosage, with the optimum performance obtained at a fiber volume fraction of 0.3%. At this dosage, the fibers are uniformly dispersed within the mortar and effectively bridge developing microcracks, delaying crack propagation and improving the integrity of the cement matrix. When the dosage is lower than 0.3%, the number of effective crack-bridging fibers is insufficient to provide significant reinforcement. Conversely, when the fiber dosage exceeds 0.3%, a slight deterioration in mechanical strength is observed. This reduction can reasonably be hypothesized to arise from reduced mixture homogeneity, where excessive fiber concentrations may promote localized fiber bundling or potentially introduce subtle micro-porosity during mixing (although air content and fresh density were not directly evaluated in this study).

To rigorously isolate the contribution of fiber thermal activation from the intrinsic influence of curing regimes on cement hydration kinetics, the mechanical evolution of the fiber-free control matrix (D1 under standard curing vs. ZD1 under steam curing) was comparatively evaluated alongside the 0.3% HSF composites (D3 vs. ZD3).

From a cement chemistry standpoint, elevated-temperature steam curing primarily accelerates early-age hydration kinetics, promoting rapid initial strength development at 7 days (as evidenced in Figure 15, where the 7-day flexural and compressive strengths of the plain mortar increased from 9.3 to 9.6 MPa and 53.6 to 56.8 MPa, respectively). From 7 to 28 days, hydration continued steadily under both regimes. However, because standard curing allows more gradual and continuous hydration at ambient temperature, the 28-day mechanical properties of the steam-cured plain mortar (ZD1: 10.6 MPa flexural, 68.5 MPa compressive) were measurably lower than those of the standard-cured control (D1: 11.2 MPa flexural, 73.0 MPa compressive).

In contrast, incorporating 0.3% HSFs exhibited a distinctly different response. While the 0.3% HSF mortar cured at 20 °C (D3) achieved 28-day flexural and compressive strengths of 12.7 MPa and 75.5 MPa through passive bridging, the same mixture subjected to 70 °C steam curing (ZD3) reached 13.2 MPa (+24.5% over ZD1) and 77.8 MPa (+13.6% over ZD1). Unlike the plain mortar where steam curing led to lower 28-day strength compared to standard curing, the HSF composite developed higher 28-day strength under steam curing than under standard curing. This net enhancement is consistent with the proposed mechanism, in which the thermal activation of the fibers generates a calculated recovery stress of ~13.7 MPa that is inferred to provide a localized confining effect around the fibers, over and above the effect of matrix hydration. This interpretation is not, however, a direct verification of matrix pre-compression: the recovery stress was obtained for a continuous bundle, and the POM comparison was not a single-variable experiment (Section 3.2.3).

**Figure 16:**
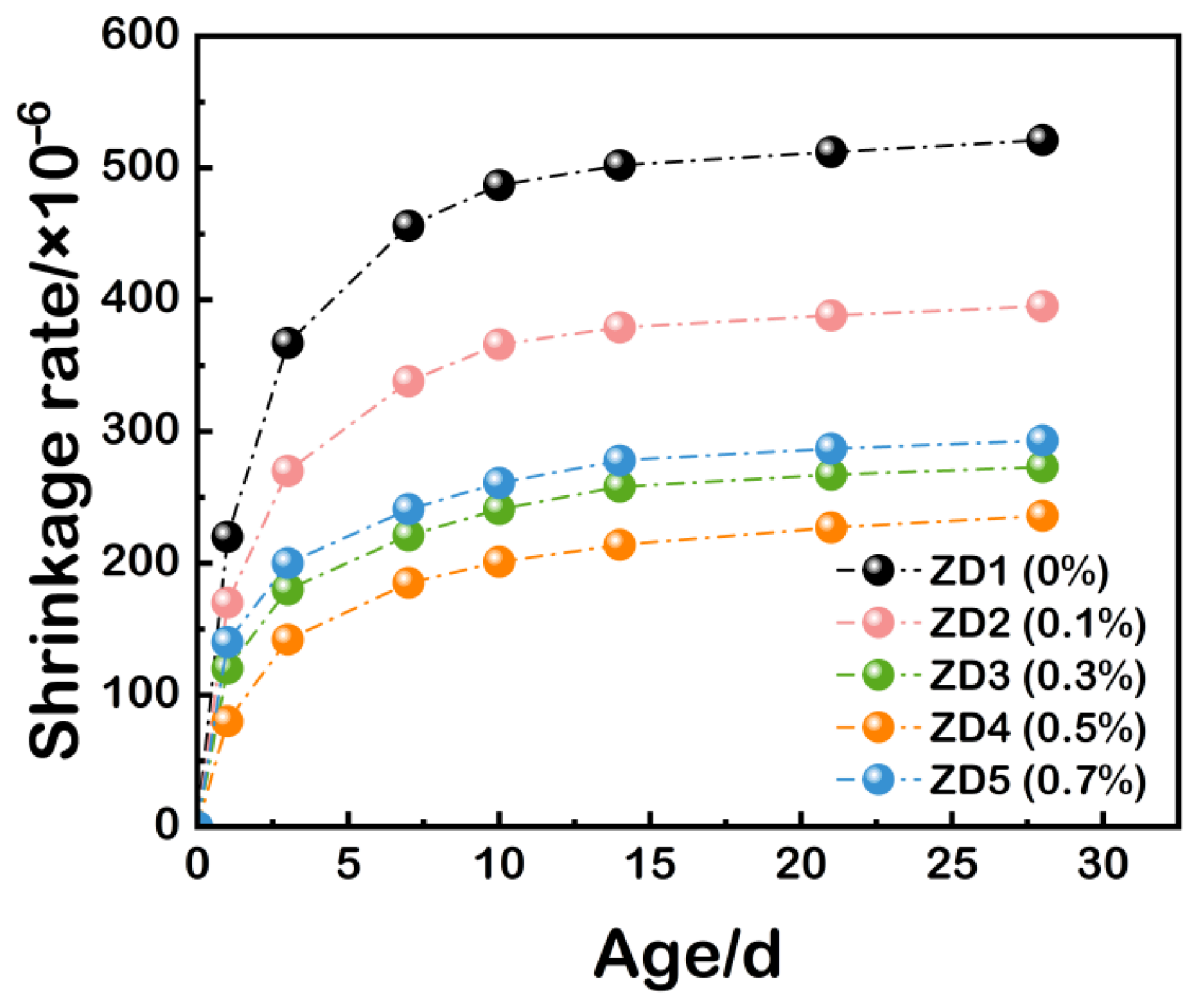
Drying shrinkage evolution of steam-cured mortar specimens with different fiber dosages (ZD series, 70 °C steam curing; ZD3 = 0.3% and ZD4 = 0.5% fiber content; the lowest absolute 28-day shrinkage was obtained at 0.5%; baseline t = 0 recorded immediately after cooling to 20 °C; n = 3).

The effect of fiber dosage on drying shrinkage is shown in Figure 16. Compared with the mortar without fibers (ZD1), all HSF-reinforced mortars showed lower drying shrinkage. Within the ZD series the lowest absolute 28-day shrinkage was recorded at 0.5% (ZD4, ~220 με, i.e., ~57% below ZD1), so the shrinkage resistance does not deteriorate between 0.3% and 0.5%. The 0.3% dosage (ZD3) is nevertheless identified as the optimum once workability and mechanical strength are considered together with shrinkage: it restricts the 28-day shrinkage to approximately 260 με (a 49.5% reduction relative to ZD1), whereas moving from 0.3% to 0.5% buys about 8 further percentage points of shrinkage while costing flowability (Figure 14) and part of the compressive strength (Figure 15). In other words, the marginal shrinkage benefit per unit fiber content decreases beyond 0.3%, so that the best overall performance is obtained at 0.3% rather than at the dosage that gives the lowest absolute shrinkage. It should also be made clear that the 233 ± 11 με value (54.7% reduction) discussed later in Section 3.2.3 comes from a separate validation batch (M3 vs. M1) cast concurrently with the POM control group, and does not belong to the ZD series. The improvement can be attributed to crack bridging combined with the thermally activated recovery of the fibers: during steam curing the fibers generate a recovery stress that restrains shrinkage deformation, and the distributed fibers bridge microcracks and delay their propagation during moisture loss.

The results in Figure 13 and Figure 16 reflect free drying shrinkage under unconstrained conditions, whereas in a restrained structure drying shrinkage is the fundamental driving force for tensile stress accumulation. Although direct restrained cracking tests (such as ring tests or cracking frame tests) were not performed in the present mortar evaluation, the substantial ~50–55% reduction in free shrinkage achieved across both experimental series, combined with the enhanced flexural strength and bridging load-bearing capacity, indicates a favorable potential to reduce the driving force for early shrinkage-induced cracking during service.

#### 3.2.3. Comparative Performance Between Non-Shrinkable and Heat-Shrinkable Fibers Under Standard and Steam Curing

To further investigate the potential contribution of thermally activated contraction to the performance of HSF-reinforced mortar, a comparative evaluation was conducted using polyoxymethylene (POM) fibers as a non-heat-shrinkable reference. The POM fibers were selected to provide a benchmark for conventional fiber reinforcement without the intended thermal contraction response of HSFs. A dedicated validation batch of mortar specimens (designated as series M: M1 for the plain control, M2 for the POM-reinforced mortar, and M3 for the mortar containing 0.3% HSFs) was independently prepared and cured concurrently under the same conditions. Both fiber types had the same nominal length of 12 mm and diameter of 200 μm and were incorporated at a target volume fraction of 0.3%. Considering their different densities (0.93 g/cm^3^ for HSFs and 1.41 g/cm^3^ for POM fibers), the corresponding fiber additions were 1.89 g and 2.87 g per batch, respectively (Table 5).

Despite the matched nominal geometry and target volume fraction, the two fiber types differed in tensile strength, elastic modulus, surface topography, and surface treatment. Specifically, the HSFs had a tensile strength of approximately 680 MPa and an elastic modulus of approximately 10.3 GPa, together with a micro-embossed and silane-functionalized surface, whereas the POM fibers had approximately 880 MPa and 8.8 GPa and retained a smooth, untreated surface. These differences may influence fiber–matrix interfacial bonding, stress transfer, and load-bearing behavior independently of thermal contraction. Therefore, the POM comparison is interpreted as an engineering-level comparison between two fiber-reinforcement systems rather than a strictly single-variable experiment. The results are used to assess whether thermally activated contraction may contribute to the observed performance differences, while recognizing that its independent contribution cannot be isolated from the effects of other fiber properties in the present experimental design. All mixtures were prepared at a constant water-to-binder ratio of 0.30 and a sand-to-binder ratio of 2.0 and were evaluated under standard curing at 20 °C and steam curing at 70 °C.

**Figure 17:**
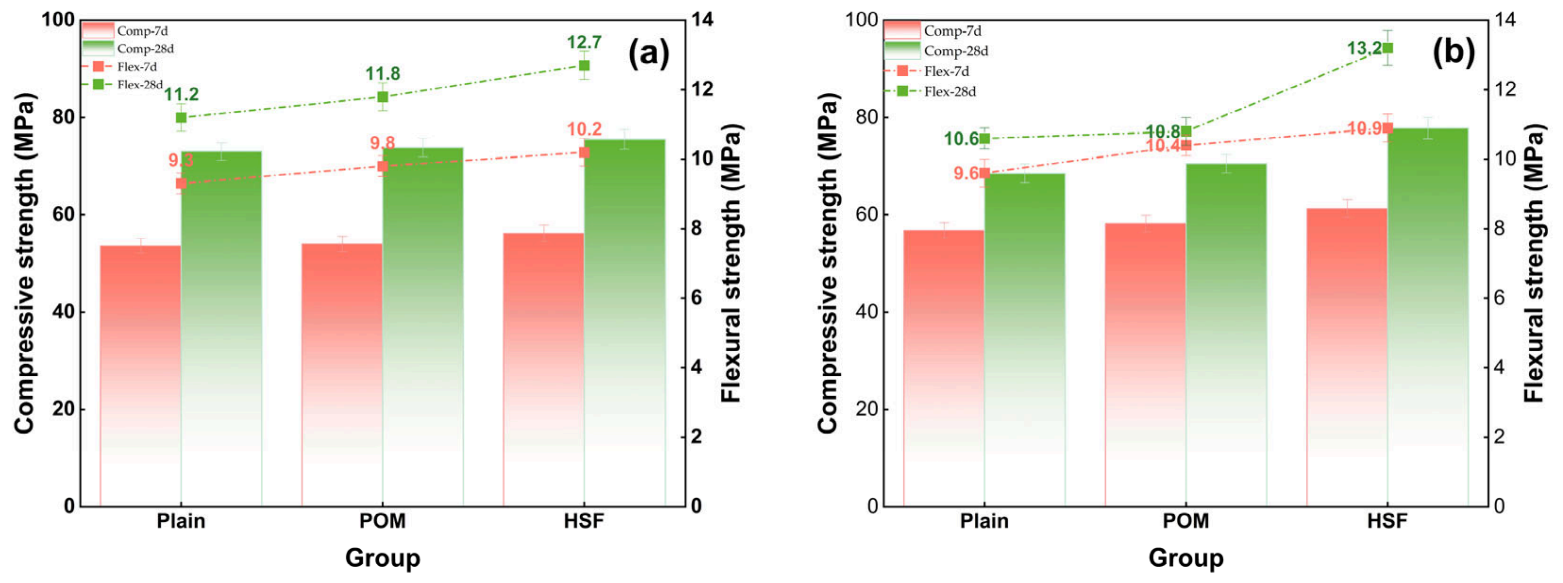
Compressive and flexural strengths of mortar reinforced with different fiber varieties under (**a**) Standard curing (20 °C) and (**b**) Steam curing (70 °C) (flexural: n = 3; compressive: n = 6; error bars represent ±1 SD).

The compressive and flexural strength development of the plain mortar, POM-reinforced mortar, and HSF-reinforced mortar under both standard curing (20 °C) and steam curing (70 °C) is presented in Figure 17.

As shown, for the unreinforced plain control (M1), steam curing promoted early-age strength gain, achieving 7-day flexural and compressive strengths of 9.6 MPa and 56.8 MPa, respectively. Over the long term (at 28 days), hydration continued and strengths increased to 10.6 MPa and 68.5 MPa; however, these 28-day values remained measurably lower than those under standard curing (11.2 MPa and 73.0 MPa) due to steam-induced microstructural coarsening.

Incorporating non-heat-shrinkable POM fibers (M2) provided passive bridging, resulting in 28-day flexural and compressive strengths of 10.8 MPa and 70.5 MPa under steam curing (Figure 17b). Under steam curing, the HSF-reinforced mortar (M3) achieved a 28-day flexural strength of 13.2 MPa, representing increases of 24.5% relative to the plain control (M1) and 22.2% relative to the POM-reinforced mortar (M2). Its compressive strength reached 77.8 MPa, compared with 68.5 MPa for M1 and 70.5 MPa for M2. These results show that the HSF-containing mortar exhibited improved mechanical performance under the tested conditions. The differences are consistent with a potential contribution from thermally activated fiber contraction. However, because the HSF and POM fibers differed in intrinsic mechanical properties and surface characteristics, the observed improvements cannot be attributed exclusively to thermal activation. The POM comparison therefore provides a useful reference for evaluating the performance of the two fiber systems, but it does not quantitatively isolate the independent contribution of thermally induced contraction from other fiber-related effects.

**Figure 18:**
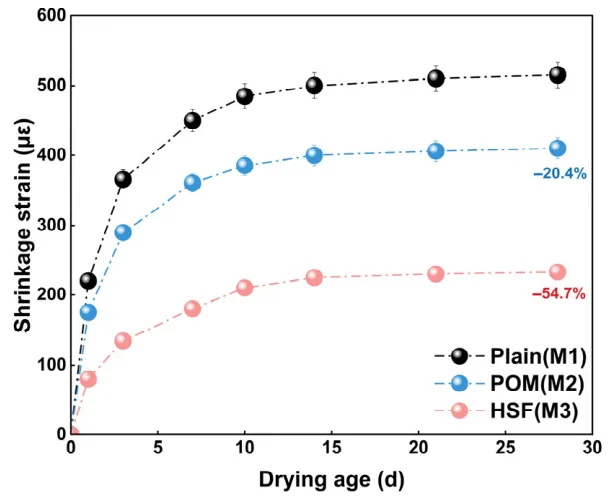
Drying shrinkage evolution of steam-cured mortar specimens reinforced with different fiber varieties (70 °C steam curing; baseline t = 0 recorded immediately after cooling to 20 °C; n = 3; error bars represent ±1 SD).

Figure 18 presents the evolution of free drying shrinkage in the dedicated validation series (M1–M3) under 70 °C steam curing over 28 days. The plain control mortar (M1) exhibited a 28-day drying shrinkage strain of 515 ± 18 με. Incorporating non-heat-shrinkable POM fibers (M2) reduced the strain to 410 ± 15 με, corresponding to a 20.4% reduction relative to M1. The HSF-reinforced mortar (M3) reached 233 ± 11 με, corresponding to a 54.7% reduction relative to M1. These results indicate that, in this validation series, the HSF-containing mortar achieved a greater reduction in free drying shrinkage than the POM-containing mortar.

The greater shrinkage reduction observed for M3 may be associated with the thermally activated contraction of HSFs, in combination with differences in fiber mechanical properties and fiber–matrix interfacial characteristics. However, because these factors were not independently controlled, their respective contributions cannot be quantitatively separated in the present study. The proposed mechanism is therefore interpreted as a plausible explanation rather than a directly verified causal relationship. Furthermore, because the drying shrinkage measurements were conducted under unrestrained conditions, they do not directly quantify the reduction in restrained tensile stress or establish the extent to which cracking is mitigated under restrained conditions.

## 4. Microstructural Characterization and Mechanism Analysis

### 4.1. SEM Characterization of the Fiber–Matrix Interface

**Figure 19:**
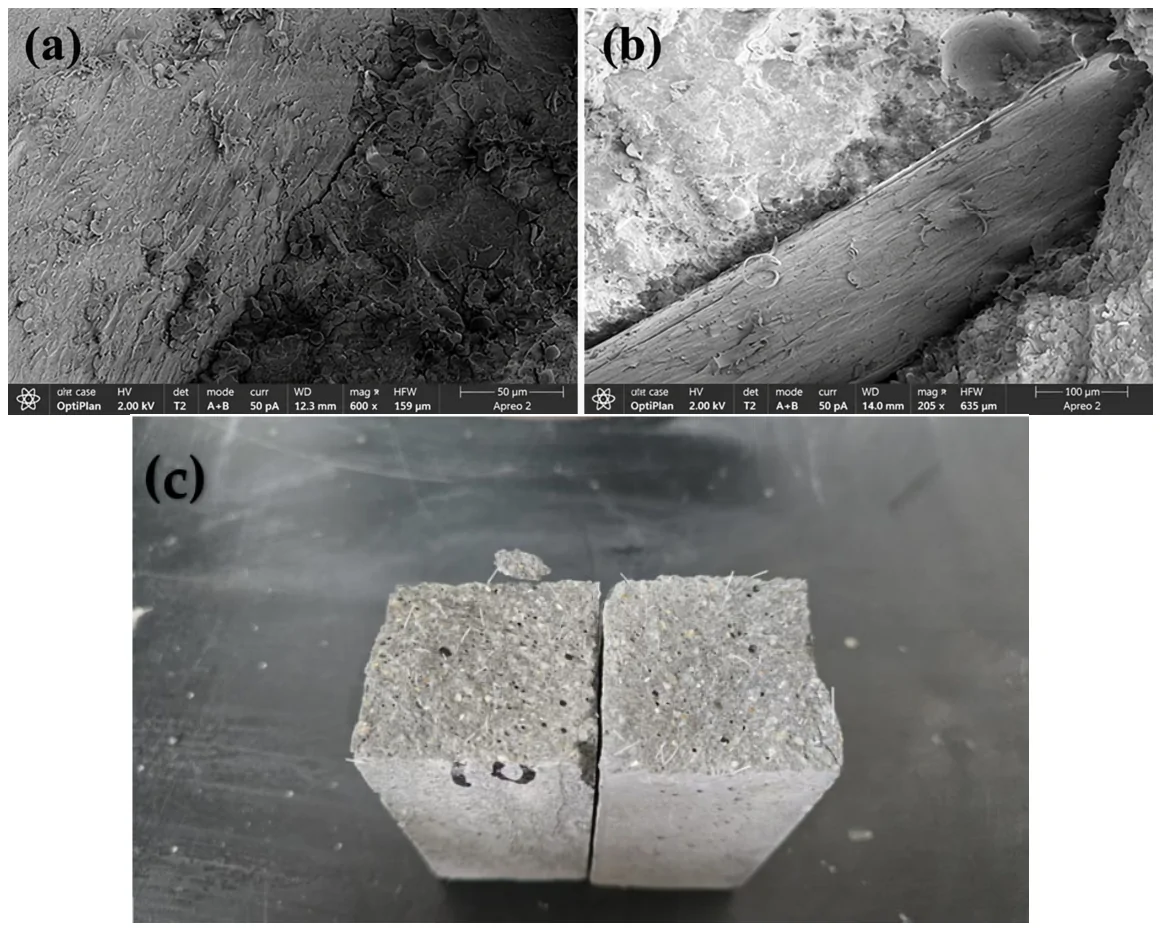
Multi-scale interfacial characterization of the fiber–matrix system under steam-curing conditions: (**a**) fiber surface morphology and matrix adherence by FE-SEM (scale bar: 50 μm); (**b**) interfacial contact morphology between fiber and matrix (scale bar: 100 μm); (**c**) macroscopic photograph of the fracture surface exhibiting uniform fiber dispersion and crack-bridging monofilaments.

As shown in Figure 19, the SEM images reveal the microstructural features of the interfacial transition zone (ITZ) between the HSFs and the surrounding cementitious matrix under steam-curing conditions. Notably, a substantial amount of cement hydration products and mortar matrix remains firmly adhered to the fiber surface after fracture. This residual matrix provides direct visual evidence of intimate interfacial contact and micro-scale mechanical interlocking between the embossed HSFs and the surrounding cementitious paste. The cementitious matrix covers the fiber surface closely, and the interfacial transition zone (ITZ) appears dense without noticeable micro-debonding gaps.

While direct interfacial bond strength was not evaluated via single-fiber pull-out testing in this study, the observation of such tightly adhering hydration products indicates that the surface-embossing and silane functionalization successfully enhance the surface wettability of the polyolefin substrate. This compact interfacial morphology is expected to favour stress transfer across the fiber–matrix boundary when thermal contraction is triggered during steam curing, which is a prerequisite for any confining action of activated fibers. The SEM observations nevertheless document interfacial quality only; they do not demonstrate that a compressive stress field develops in the surrounding paste.

The mortar adhering to the fiber surface indicates a reliable interfacial connection. This high-quality bonding is expected to facilitate the transfer of the thermally induced recovery stress from the activated fibers to the surrounding matrix, which is a prerequisite for any internal restraint and for the crack-bridging efficiency of the HSFs; this is in good agreement with the interfacial micromechanical reinforcement and ITZ densification observed in fiber-modified concrete systems [68]. This microstructural finding is consistent with the improved macro-shrinkage resistance presented in Section 3.2.

To quantitatively assess the degree of fiber dispersion and evaluate the presence of agglomerates, a statistical cross-sectional grid-counting technique was implemented on diamond-sawn cross-sections of hardened mortar prisms (40 × 40 mm). Each cross-section was discretized into a 4 × 4 square grid (M = 16 grid units of 10 × 10 mm each). Under an optical stereomicroscope, the number of individual fiber ends ($n_{i}$) in each grid unit was counted, and the fiber dispersion coefficient (β) was calculated according to Equation (5):

(5) $$\beta =\exp\left[-\frac{1}{\bar{n}}\sqrt{\frac{\sum_{i=1}^{M}{\left(n_{i}-\bar{n}\right)}^{2}}{M}}\right]$$

where $\bar{n}$ is the average fiber count per unit area, and *M* is the total number of grid units. A β value approaching 1.0 signifies ideally uniform spatial distribution, whereas lower values indicate severe spatial segregation or agglomeration.

Three cross-sections were evaluated for each mixture. For the optimal 0.3% HSF mortar, the calculated dispersion coefficient reached β = 0.89 ± 0.03. This high dispersion index, coupled with a low coefficient of variation across grids (<12%), quantitatively validates that the special surfactant coating and optimized mixing protocol effectively suppress inter-fiber entanglement, securing uniform 3D dispersion without macro-agglomerates. In contrast, when the fiber volume fraction was increased to 0.7%, the dispersion coefficient dropped sharply to β = 0.67 ± 0.05, with individual grid fiber counts fluctuating markedly. This trend is consistent with localized fiber bundling and crowding at high dosages, which would explain the observed reductions in fresh fluidity and hardened compressive strength.

In addition to micro-scale observations, the macroscopic dispersion and bridging behavior of the HSFs were documented from the fracture surface of the prism specimens, as shown in Figure 19c. Consistent with the dense interfacial contact seen in SEM, individual monofilaments are visibly distributed throughout the mortar matrix without bundle agglomeration or dry nests. Across the flexural fracture plane, numerous fibers protrude from both split faces and remain intact, providing continuous mechanical bridging across developing cracks. This macroscopic visual evidence directly corroborates the absence of localized clustering and substantiates the enhanced post-peak structural integrity observed during mechanical testing.

### 4.2. FTIR Analysis of Chemical Structure and Interfacial Bonding

**Figure 20:**
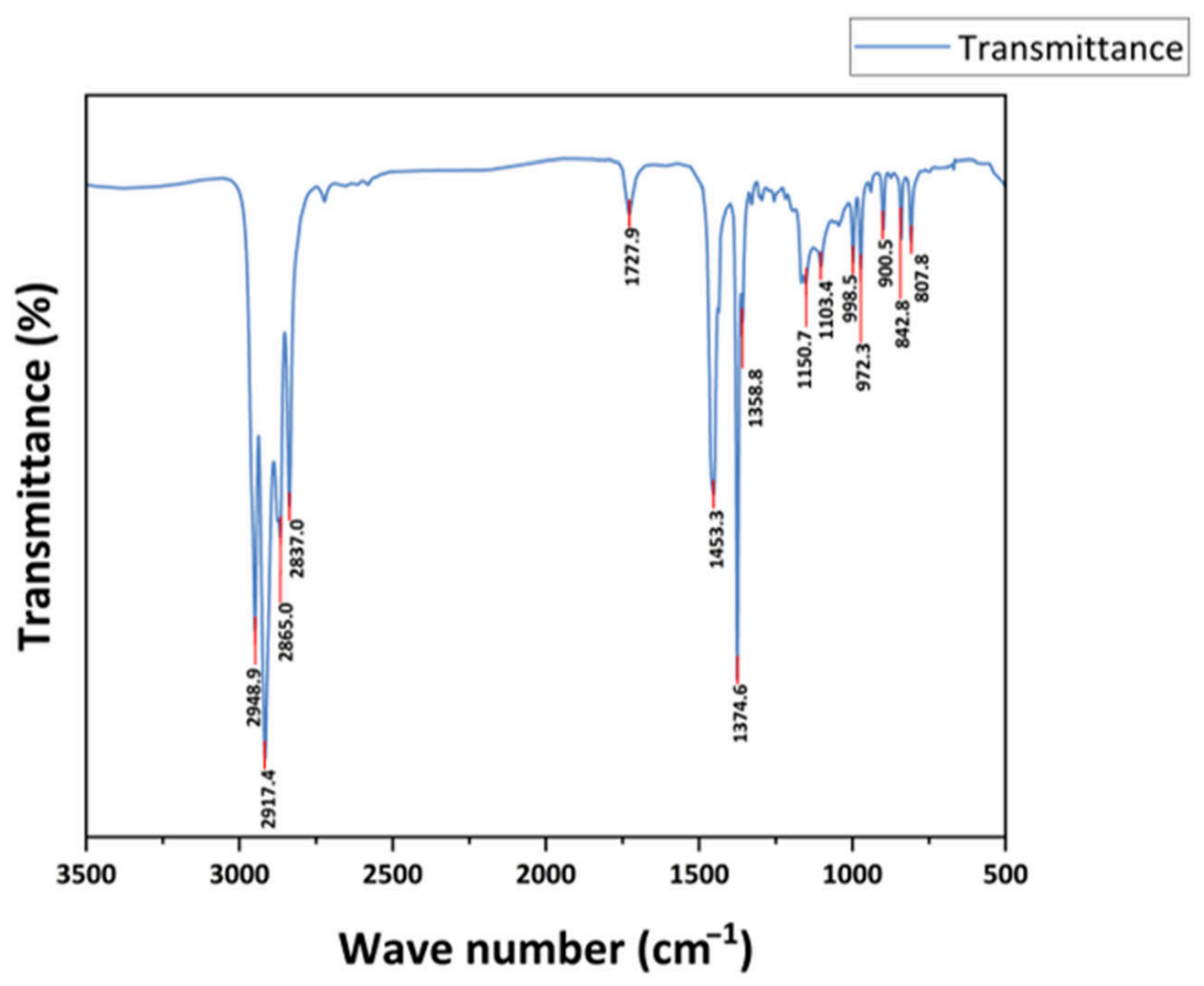
Comparative Fourier Transform Infrared (FTIR) spectra of unmodified PP/MHMWPE blend fibers and surface-modified heat-shrinkable fibers (HSFs).

To characterize the chemical structure and surface functionalization of the HSFs, Fourier transform infrared (FTIR) spectroscopy was performed. As shown in Figure 20, the spectrum exhibits characteristic absorption bands at 2948.9, 2917.4, and 2865.0 cm^−1^, corresponding to the asymmetric and symmetric stretching vibrations of C–H bonds in methyl and methylene groups. In addition, the absorption peaks at 1453.3 and 1374.6 cm^−1^ are attributed to the bending vibration and symmetric deformation of methyl groups, respectively, consistent with the characteristic molecular structure of the polypropylene/polyethylene-based matrix.

In addition to the characteristic aliphatic polyolefin hydrocarbon bands, a distinct absorption band appears at 1727.9 cm^−1^, which corresponds to the stretching vibration of carbonyl (C=O) groups associated with the grafted maleic anhydride and polar surface treatments. Furthermore, the characteristic absorption bands emerging at 1150.7 cm^−1^ and 1103.4 cm^−1^ are assigned to Si–O–C and Si–O–Si asymmetric stretching vibrations from the silane coupling agent (γ-APS). Because pristine polypropylene and polyethylene backbones consist purely of non-polar saturated C–H and C–C bonds with no inherent absorption at the specific carbonyl (~1728 cm^−1^) and siloxane (~1151 and ~1103 cm^−1^) positions, the presence of these distinctive functional bands provides clear spectroscopic evidence of successful surface grafting and functionalization.

The introduction of these polar organosilicon and oxygen-containing functional groups increases the surface polarity and chemical affinity of the inherently inert polyolefin substrate toward polar cement hydration products. While direct wettability was not quantitatively evaluated via dynamic contact-angle measurements in this study, this surface chemical functionalization, combined with the micro-embossed physical indentations, contributes to improved matrix wettability and paste adherence, supporting the dense interfacial transition zone (ITZ) observed in the SEM micrographs.

To semi-quantitatively evaluate the relative abundance of surface-introduced functional groups, a spectroscopic indexing method was applied. The bending vibration peak of the stable aliphatic polyolefin backbone at 1453.3 cm^−1^ (I_1453_) was chosen as an internal reference standard, as its intensity remains essentially unaffected by surface treatments. The relative Carbonyl Index (CI) and Silane Grafting Index (SI) were calculated according to Equations (6) and (7):

(6) $$CI=\frac{I_{1728}}{I_{1453}}$$

(7) $$SI=\frac{I_{1103}}{I_{1453}}$$

where I_1728_ and I_1103_ represent the baseline-corrected peak intensities corresponding to the carbonyl (C=O) and siloxane (Si–O–Si) functional groups, respectively.

As illustrated in Figure 20, the pristine, unmodified PP/MHMWPE blend fiber exhibits negligible absorption across these characteristic regions, showing baseline values of CI = 0.012 ± 0.003 and SI = 0.008 ± 0.002. Following surface modification and silane treatment, the modified HSF exhibits marked increases in both indices, reaching CI = 0.365 ± 0.021 and SI = 0.294 ± 0.018. Rather than serving as an absolute measure of chemical grafting yield, these spectroscopic index increments provide comparative evidence for the introduction and retention of polar organosilicon and oxygen-containing functional layers on the polyolefin fiber surface.

### 4.3. TG/DTG Analysis of Thermal Stability

**Figure 21:**
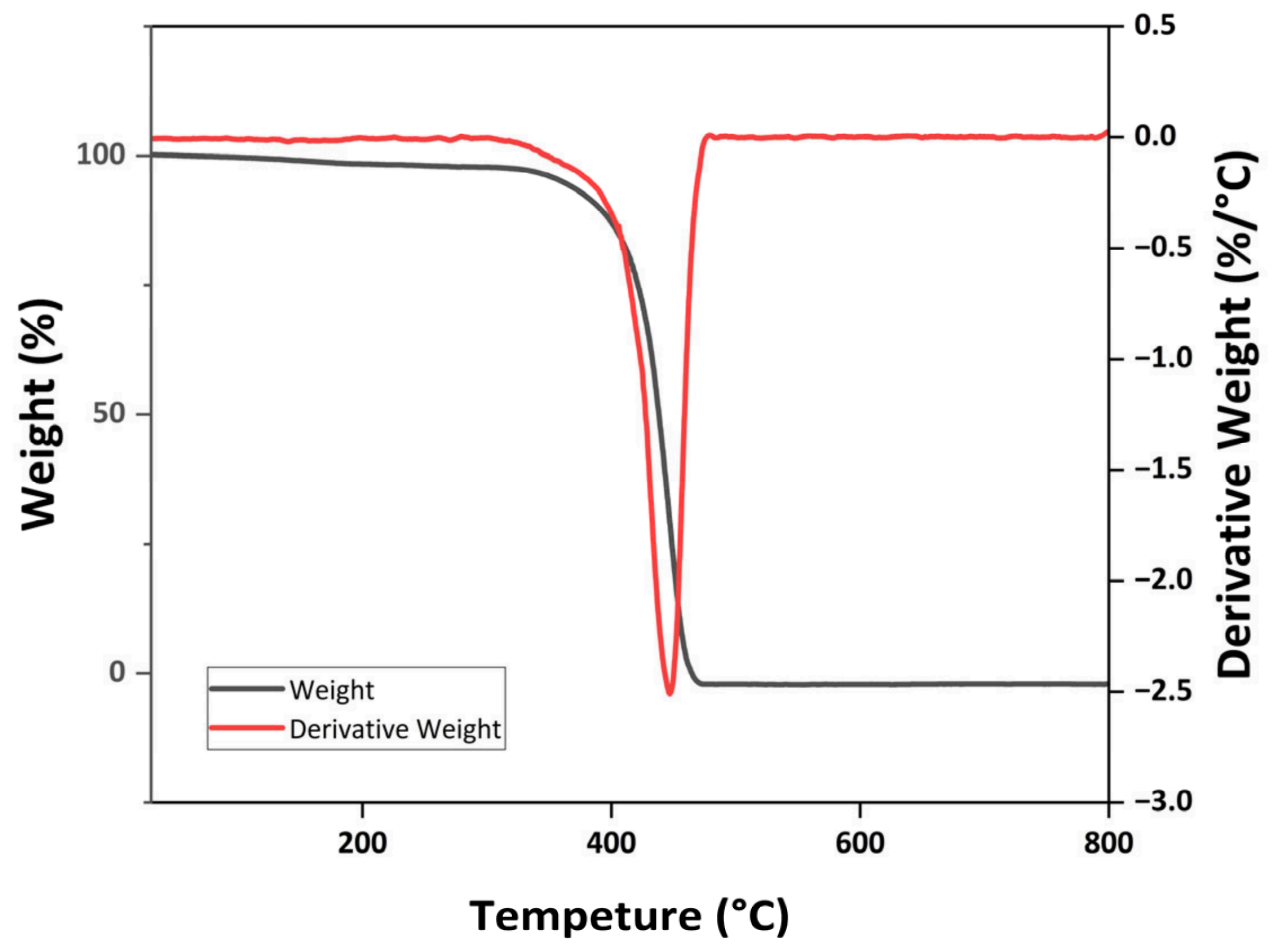
TG/DTG curves of the modified heat-shrinkable fibers.

To evaluate the thermal stability of the HSFs and their suitability for application in mass concrete, thermogravimetric (TG) and derivative thermogravimetric (DTG) analyses were performed. The corresponding TG and DTG curves are presented in Figure 21.

As shown in Figure 21, the HSFs exhibit a typical single-stage thermal degradation process. From room temperature to approximately 300 °C, the TG curve remains nearly horizontal, with negligible mass loss, while no significant peak is observed in the DTG curve. These results indicate that the HSFs possess good thermal stability within this temperature range. Considering that the thermal activation window of the HSFs is between 50 and 90 °C, the fibers remain thermally stable throughout the thermal activation process without undergoing detectable thermal decomposition.

A rapid decrease in mass occurs when the temperature exceeds approximately 300 °C, and the maximum degradation rate is observed at around 450 °C, as indicated by the DTG peak. Thermal decomposition is essentially completed at approximately 480 °C, leaving only negligible residual mass. This degradation behavior is characteristic of polyolefin-based materials and is consistent with the polypropylene/polyethylene matrix of the HSFs.

The TG/DTG results demonstrate that the HSFs possess sufficient thermal stability for practical applications in cementitious materials. Since the temperatures generated during cement hydration are far below the onset of thermal degradation, the fibers are able to maintain their structural integrity throughout the hydration process. This stability provides a reliable basis for the thermally activated shrinkage behavior and recovery stress discussed in the previous sections.

### 4.4. Crystallinity Evolution of HSFs

**Figure 22:**
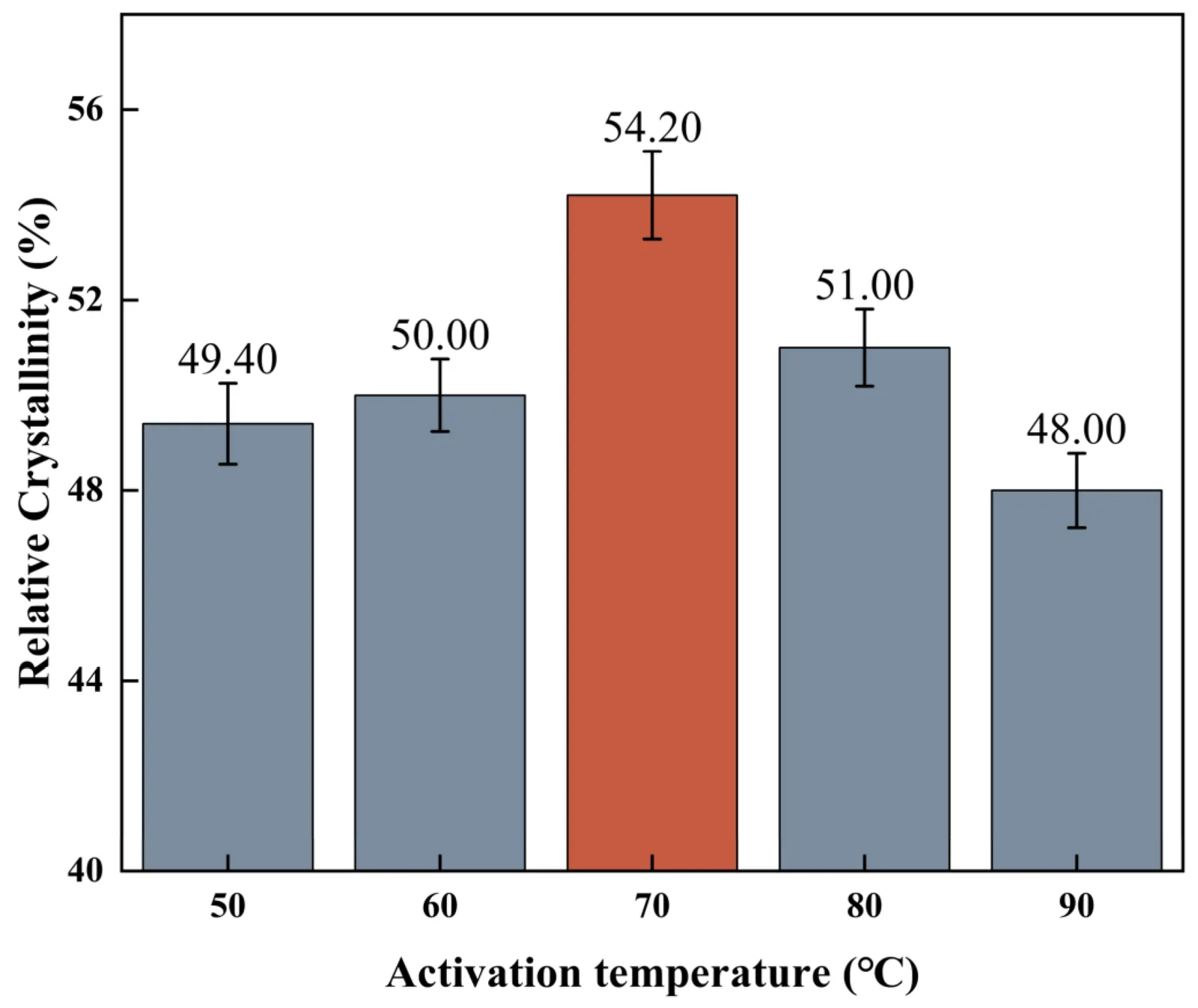
Relative crystallinity of heat-shrinkable fibers after thermal exposure at different temperatures (the red bar highlights the optimal activation temperature of 70 °C achieving peak crystallinity; determined via DSC, n = 3; error bars represent ±1 standard deviation).From an engineering and material performance standpoint, 70 °C serves as the preferred activation regime for the composite evaluations. At this temperature, the HSFs achieve their maximum crystalline perfection and highest room-temperature elastic modulus (11.5 GPa), while simultaneously developing a substantial recovery stress of 13.72 MPa (92.8% of the peak value observed at 90 °C). Thermal exposure above 70 °C (up to 90 °C) does not induce harmful thermal degradation, as the fibers stably retain an elastic modulus above 10.2 GPa; however, 70 °C gives the highest stiffness and the most stable crystallinity of the temperatures studied. Furthermore, 70 °C aligns closely with the core thermal plateau naturally reached during the hydration of mass concrete structures (60–80 °C), securing efficient interfacial stress transfer under realistic curing conditions.

To further clarify the microstructural origin of the thermomechanical behavior of the HSFs, the relative crystallinity of the fibers after thermal treatment at different temperatures was analyzed. As shown in Figure 22, the relative crystallinity exhibits an inverted U-shaped trend with increasing activation temperature, increasing from 49.40 ± 0.85% at 50 °C to a maximum value of 54.20 ± 0.92% at 70 °C, followed by a gradual decrease to 48.00 ± 0.78% at 90 °C.

The increase in crystallinity between 50 and 70 °C can be attributed to thermally induced molecular rearrangement and secondary crystallization. Moderate thermal activation enhances the mobility of polymer chain segments, allowing previously constrained chains to rearrange into a more ordered crystalline structure. Consequently, crystal perfection is improved, resulting in a higher degree of crystallinity.

When the activation temperature exceeds 70 °C, the relative crystallinity gradually decreases. At higher temperatures, excessive molecular mobility reduces the stability of the ordered crystalline regions and partially disrupts the regular packing of polymer chains. As a result, the crystalline order decreases, leading to a reduction in the measured relative crystallinity.

The evolution of crystallinity is in good agreement with the changes in the elastic modulus discussed in Section 3.1.1. Both properties reach their maximum values at approximately 70 °C, indicating that moderate thermal activation promotes the formation of a more ordered and mechanically stable microstructure. The improved crystalline structure contributes to higher stiffness and facilitates more effective transfer of the thermally generated recovery stress from the fibers to the surrounding cementitious matrix. Conversely, excessive activation temperatures reduce crystalline order and slightly decrease the mechanical performance of the HSFs.

Overall, the crystallinity analysis elucidates the microstructural origin of the temperature-dependent thermomechanical behavior of the HSFs. It is important to distinguish the performance criteria when defining the activation optimum:

From a microstructural and stiffness perspective, approximately 70 °C represents the optimal activation temperature, where secondary crystallization and molecular ordering culminate in peak relative crystallinity (54.20%) and maximum room-temperature elastic modulus (11.5 GPa). Thermal exposure beyond 70 °C induces partial reorganization of less stable crystalline regions, leading to minor decreases in crystallinity and modulus.

From an actuation perspective, the unconstrained shrinkage ratio (2.80%) and isometric recovery stress (14.79 MPa) continue to increase up to 90 °C, driven by the accelerated entropic recoiling of oriented amorphous chains at higher thermal energies.

### 4.5. Limitations and Future Perspectives

Although the current study systematically demonstrates the thermomechanical properties of HSFs and their effectiveness in enhancing mortar strength and reducing drying shrinkage, several experimental and analytical limitations should be recognized:

(1) Unrestrained vs. Restrained Conditions: The drying shrinkage measurements herein were performed under free, unrestrained conditions. While lowering free shrinkage fundamentally decreases the primary driving potential for stress build-up, direct restrained cracking evaluations (such as restrained ring cracking tests or temperature-stress testing machines) were not conducted in this work. Consequently, the actual cracking age, degree of stress relaxation, and true cracking resistance under structural restraint remain to be directly quantified.
(2) Flexural Strength vs. Toughness: The flexural test focused primarily on the peak modulus of rupture. Comprehensive load–deflection (P−δ) response curves and post-cracking residual strength parameters were not recorded; therefore, the reported flexural improvements reflect enhanced peak load-carrying capacity rather than a direct quantification of post-cracking fracture toughness or energy absorption.
(3) Inferred Prestressing Mechanism: The concept of internal matrix pre-compression is inferred from single-bundle recovery stress tests and microstructural observations rather than direct micro-scale stress sensor measurements within the paste. Further multi-axial restraint testing and multi-scale finite element micromechanical simulations will be pursued in future investigations to establish direct quantitative links between fiber activation and matrix stress relief. Accordingly, the calculated recovery stress, the reduced free drying shrinkage and the improved peak flexural strength reported here are not presented as direct verification of cracking resistance under structural restraint; restrained ring or cracking-frame tests, together with post-cracking load–deflection measurements, are required before such a claim can be made.

## 5. Conclusions

Based on the experimental investigation, microstructural characterization, and engineering validation presented in this study, the following conclusions can be drawn:

(1) High-modulus heat-shrinkable fibers (HSFs) capable of thermal activation were successfully developed for mitigating shrinkage in high-strength cementitious composites. The HSFs exhibited a thermal activation range of 50–90 °C, tensile strength exceeding 600 MPa, and an elastic modulus above 9 GPa. Their thermal shrinkage behavior was primarily governed by the activation temperature, while the initial fiber length had a negligible influence on the shrinkage ratio.
(2) The thermally activated shrinkage and recovery stress of the HSFs were systematically characterized under laboratory conditions. Both the free shrinkage ratio and the calculated isometric recovery stress increased monotonically with activation temperature within the 50–90 °C window (Tonset = 50 °C), reaching peak values of 2.80% and 14.79 MPa at 90 °C, respectively. Concurrently, the fibers remained thermally stable up to 300 °C. The relative crystallinity reached a maximum (54.20%) at 70 °C, corresponding to peak room-temperature elastic modulus (11.5 GPa). These thermomechanical responses are attributed to the synergistic effect of radiation-induced crosslinking, molecular chain orientation, and secondary crystallization during thermal exposure.
(3) Within an optimal volume fraction range (up to 0.3%), HSF incorporation significantly reduced drying shrinkage and improved flexural strength without compromising compressive strength. Specifically, mortar with 12 mm fibers at 0.3% volume fraction exhibited optimal overall performance, achieving a 28-day flexural strength of 13.2 MPa and compressive strength of 77.8 MPa under steam curing, accompanied by reductions in 28-day drying shrinkage of ~49.5% in the dosage-screening (ZD) series and 54.7% in the independent validation (M) series relative to their respective controls. The lowest absolute 28-day shrinkage (~220 με) was obtained at 0.5% fiber content, but this dosage also reduced fluidity and slightly lowered compressive strength; consequently, 0.3% is recommended as the optimum when workability and strength are taken into account. The significant shrinkage reduction is consistent with the proposed mechanism that fiber crack-bridging, coupled with thermally induced recovery stress, provides internal restraint that may help counteract early-age contraction deformation. Steam curing raised these strengths further, whereas it lowered those of the plain matrix, which again points to a contribution from thermal activation.

## Figures and Tables

**Table 1:** Chemical compositions of the cementitious materials (wt.%).

Chemical Composition (wt.%)	Cement (P·II 52.5)	Fly Ash (I-FA)	Slag (GGBFS)
SiO_2_	19.83	52.40	36.21
Al_2_O_3_	4.38	28.15	12.36
Fe_2_O_3_	3.27	5.80	8.51
CaO	64.08	4.10	34.87
MgO	1.25	1.20	1.68
SO_3_	2.21	1.56	0.35
K_2_O	0.84	1.10	0.97
Na_2_O	0.16	0.40	0.68
TiO_2_	0.25	—	—
Loss on ignition (LOI)	1.45	3.20	1.10

Note: “—“ indicates not detected or not applicable.

**Table 2:** Physical properties of the cementitious materials.

Physical Properties	Cement (P·II 52.5)	Fly Ash (I-FA)	Slag (GGBFS)
Density (g/cm^3^)	3.16	2.25	2.90
Specific surface area (m^2^/kg)	340	442	463
Water demand ratio (%)	—	93	—
Fineness (45 μm sieve residue, %)	—	14.4	—

Note: “—“ indicates not determined or not applicable.

**Table 3:** Experimental mix ratios of cement mortar with different heat-shrinkable fiber lengths.

No.	Cement (g)	Slag Powder (g)	Fly Ash (g)	Water (g)	Fiber Mass (m_f_, g)	Medium Sand (g)	Admixture (g)	Fiber Length (mm)
C1	300	75	125	150	1.89	1000	2	8
C2	300	75	125	150	1.89	1000	2	12
C3	300	75	125	150	1.89	1000	2	16
C4	300	75	125	150	1.89	1000	2	20

Note: The fiber mass per batch was calculated as m_f_ = V_batch_ × V_f_ × ρ_f_, with ρ_f_ = 0.93 g/cm^3^, V_batch_ ≈ 677.4 cm^3^ (the compacted batch volume used for the dosage calculation) and V_f_ the fiber volume fraction. In this series V_f_ was 0.3% (1.89 g per batch) and only the fiber length was varied.

**Table 4:** Experimental mix ratios of cement mortar with different heat-shrinkable fiber dosages.

No.	Cement (g)	Slag Powder (g)	Fly Ash (g)	Water (g)	Fiber Mass (m_f_, g)	Medium Sand (g)	Admixture (g)	Fiber Length (mm)
D1	300	75	125	150	0	1000	2	12
D2	300	75	125	150	0.63	1000	2	12
D3	300	75	125	150	1.89	1000	2	12
D4	300	75	125	150	3.14	1000	2	12
D5	300	75	125	150	4.40	1000	2	12

Note: The admixture dosage (2.0 g, corresponding to 0.4 wt.% of total binder) refers to the mass of the commercial polycarboxylate ether (PCE) aqueous solution (40% solid content) as supplied. For a batch volume of 677.4 cm^3^ and a fiber density of 0.93 g/cm^3^, the fiber volume fractions of 0%, 0.1%, 0.3%, 0.5% and 0.7% correspond to fiber masses of 0, 0.63, 1.89, 3.14 and 4.40 g per batch, respectively.

**Table 5:** Physical properties of the fibers and mix proportions of the mortar ($V_{f}\approx 0.3\%$).

Group	Fiber Type	Density (g/cm^3^)	Diameter (μm)	Length (mm)	Tensile Strength (MPa)	Elastic Modulus (GPa)	Surface Condition	Fiber Mass (m_f_, g)	Volume Fraction (V_f_, %)	Thermal Shrinkage
M1	Plain mortar	—	—	—	—	—	—	0	0	—
M2	POM fiber	1.41	200	12	≈880 *	≈8.8	Smooth, untreated	2.87 *	0.30	None
M3	HSF	0.93	200	12	≈680	≈10.3 **	Embossed, silane-grafted	1.89	0.30	2.8% at 90 °C

Note: w/b = 0.30; s/b = 2.0. * Manufacturer’s data (technical data sheet). ** Room-temperature modulus measured after exposure to 90 °C and cooling; the peak value of 11.5 GPa was obtained at 70 °C (Figure 7). Fiber mass per batch: $m_{f}=V_{batch}\times V_{f}\times \rho_{f}$, with V_batch_ = 677.4 cm^3^.

## Data Availability

The original contributions presented in this study are included in the article. Further inquiries can be directed to the corresponding authors.

## Abbreviations

The following abbreviations are used in this manuscript:

HSF	Heat-Shrinkable Fibers
PP	Polypropylene
PVA	Polyvinyl alcohol
MHMWPE	Medium-high molecular weight polyethylene
ECC	Engineered cementitious composites
FA	Fly ash
GGBFS	Ground granulated blast furnace slag
XRF	X-ray fluorescence
mPP	Metallocene polypropylene
SCMs	Supplementary cementitious materials
PP-g-MAH	Maleic anhydride grafted polypropylene
iPP-b-PE	isotactic polypropylene-block-polyethylene
